# Nanoscale motion of organic π-conjugated molecules: exploring van der Waals forces, friction, and quantum effects

**DOI:** 10.1039/d5nh00414d

**Published:** 2025-09-09

**Authors:** Anton Tamtögl, Marco Sacchi

**Affiliations:** a Institute of Experimental Physics, Graz University of Technology Graz Austria tamtoegl@tugraz.at; b School of Chemistry and Chemical Engineering, University of Surrey Guildford GU2 7XH UK

## Abstract

The single-molecule dynamics of π-conjugated organic molecules on surfaces is fundamental for applications ranging from catalysis to molecular electronics. Adsorption and diffusion, in particular of organic aromatics, are typically driven by van der Waals forces, energy dissipation in terms of friction, and quantum effects, making them ideal for probing surface energy landscapes. However, their fast motion at thermal equilibrium poses experimental challenges. Recent advances have provided unprecedented insights into the diffusion mechanisms of several organic molecules on metallic and graphitic surfaces. These studies reveal a spectrum of motion, from ballistic transport to Brownian diffusion, influenced by surface symmetry, molecular size, charge transfer, and molecular degrees of freedom. Notably, friction at 2D material interfaces can be exceptionally low, leading to superlubricity – a phenomenon which highlights the role of atomic-scale interactions in determining energy dissipation and molecular mobility. We review experimental and computational techniques capturing diffusion at atomic length scales, highlighting how density functional theory and molecular dynamics complement experimental findings. Despite recent advances, key questions remain, such as how friction varies across different surfaces and how external factors affect mobility. Understanding these interactions is essential for controlling molecular assembly and surface functionalisation: controlling diffusion and dissipation at the nanoscale may enable self-assembled nanostructures, where controlled molecular motion drives highly ordered surface architectures. Finally, beyond technological applications, surface diffusion is also critical in astrochemistry, where it influences the formation of complex organic molecules.

## Abstract 2

We review how the motion of π-conjugated organic molecules on surfaces is shaped by physicochemical factors such as friction and molecule–surface interactions, revealing mechanisms that govern diffusion, dissipation, and nanoscale assembly.

## Graphical abstract

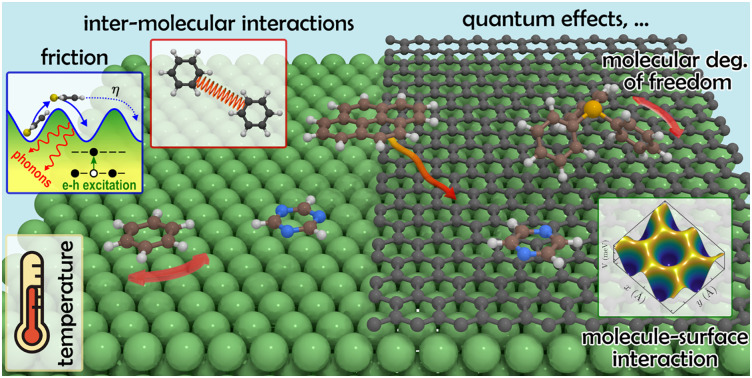

## Introduction

Understanding the single-molecule dynamics of π-conjugated organic molecules on surfaces is of critical importance for a range of applications, including catalysis, crystal growth, molecular electronics, and nanotechnology.^1–7^ Surface diffusion governs many of these processes, where the motion of adatoms or molecules determines the kinetics of reactions, self-assembly, or layer formation.^8–11^ Typically, the diffusion rate depends on transitions across energy barriers shaped by the potential energy surface and the coupling to substrate excitations, such as phonons or electron–hole pairs. The self-assembly of molecules into ordered supramolecular structures enables a bottom-up strategy for nanoscale fabrication, offering tunable surfaces for use in sensors, coatings, photon harvesting, and molecular recognition.^1,11–15^ The resulting architectures emerge from a subtle interplay between molecule–substrate and intermolecular interactions. Recent advances in experimental techniques have enabled detailed studies of molecular diffusion on metal surfaces, where stronger molecule–substrate interactions make scanning tunnelling microscopy (STM) feasible.^16–18^

However, our understanding of such dynamics on weakly interacting, inert substrates like graphite/graphene and hexagonal boron nitride (h-BN) remains limited.^11,14,19^ On these two-dimensional (2D) materials, van der Waals (vdW) interactions dominate, leading to fast (pico- to nanosecond) molecular motion and even at low temperature, molecular motion can be tip-induced in STM measurements.^20–22^ Hydrocarbons and aromatic molecules display diverse behaviours on graphitic surfaces. Their motion is shaped by several factors, among which are molecular size, geometry, charge transfer, surface symmetry, and internal degrees of freedom. Controlling these factors may allow us to tailor molecular motion at the nanoscale. For instance, at 2D material interfaces, friction can be remarkably low, a phenomenon termed superlubricity,^23–25^ which highlights the importance of energy dissipation mechanisms at the nanoscale.

In this context, we review recent advances ranging from benzene to heterocyclic aromatics and polycyclic aromatic hydrocarbons (PAHs), and we conclude with an outlook on the diffusion of other, often larger and more complex π-conjugated organic molecules. Recent experimental developments have enabled insight into the diffusion mechanisms of such molecules on metal and graphitic surfaces. These studies reveal that hydrocarbons can exhibit a range of motion, from ballistic transport to Brownian motion, influenced by several of the aforementioned factors. Moreover, the adsorption and diffusion of these systems are governed by vdW interactions, electronic friction, and quantum effects, making them ideal systems to probe the nanoscopic energy landscape at surfaces. The adsorption of benzene and PAHs on 2D materials also serves as a benchmark system for refining computational approaches. They are frequently employed to validate dispersion-corrected density functional theory (DFT), molecular dynamics (MD)^26^ and the well-characterised interaction of benzene with metal surfaces makes it an ideal platform for assessing the accuracy of such theoretical methods.^27–30^

Beyond fundamental research, understanding the dynamics of organic π-conjugated molecules on surfaces is crucial for advancing the controlled synthesis and functionalisation of 2D materials. The adsorption behaviour, mobility, and interaction of such molecules significantly influence the morphology, crystallinity, and doping characteristics of the resulting materials.^31–33^ Investigations into physisorbed aromatics reveal their role in modulating surface properties and enabling precise growth techniques such as parallel stitching,^34^ or the formation of semiconducting polymer networks.^35^ The reactivity of these species, including their capacity for metalation^36^ and covalent coupling,^37^ underpins the development of structurally defined and electronically tunable frameworks. Furthermore, recent approaches and aromatic rules for structural design underscore the need for a deeper mechanistic understanding.^38–40^ These insights extend to applications such as nanoporous sheets,^41^ conductive covalent organic frameworks,^42^ and hybrid perovskites with tailored optoelectronic properties,^43^ emphasising the pivotal role of surface-bound aromatic molecule dynamics in next-generation materials engineering.^44,45^ In parallel, surface-bound aromatics serve as active sites or precursors in heterogeneous catalysis, contributing to enhanced selectivity and activity in various catalytic systems.^46–49^ Notably, the interaction of such molecules with surfaces also has implications for environmental processes, such as the adsorption of pollutants^50^ and the formation of soot particles.^51^

### Summary

In summary, understanding these interactions is essential not only for controlling molecular assembly and surface functionalisation but also for predicting energy transport pathways in complex systems. Beyond technological relevance, such knowledge is crucial for astrochemical models, where surface mobility contributes to the synthesis of complex organic molecules in interstellar environments.^52,53^

### Aromatics and π-conjugated molecules

This review focuses on the diffusion and surface dynamics of π-conjugated organic molecules for which data is available. Table 1 provides an overview of the corresponding molecules, ranging from simple aromatics to larger and more complex systems. We start with benzene (C_6_H_6_), the simplest and most well-studied aromatic molecule. Due to its planar, highly symmetric π-electron system, it serves as a model system to probe π-surface interactions and benchmark theoretical approaches. Closely related compounds, such as borazine, which is used as a precursor in the growth of h-BN^54,55^ share similar adsorption characteristics due to their aromatic nature and symmetric structure.

**Table 1:** Overview of specific types of organic molecules for which diffusion and surface mobility are discussed in the review. The right-most column illustrates a representative molecular geometry from each group

Molecule and chemical formula		Example
Benzene		
	C_6_H_6_	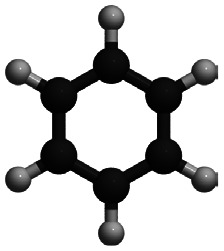
Heterocyclic organic rings		
Pyrazine	C_4_H_4_N_2_	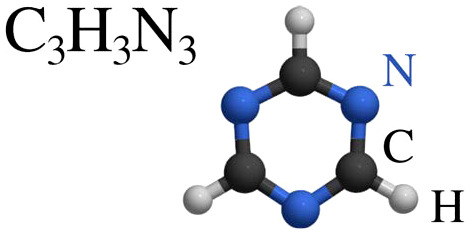
s-Triazine	C_3_H_3_N_3_	
5-Membered rings		
Cyclopentadienyl	C_5_H_5_	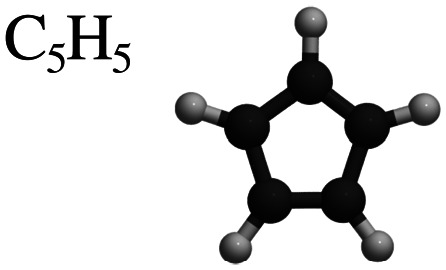
Pyrrole	C_4_H_5_N	
Polyaromatic hydrocarbons (PAHs)		
Naphthalene	C_10_H_8_	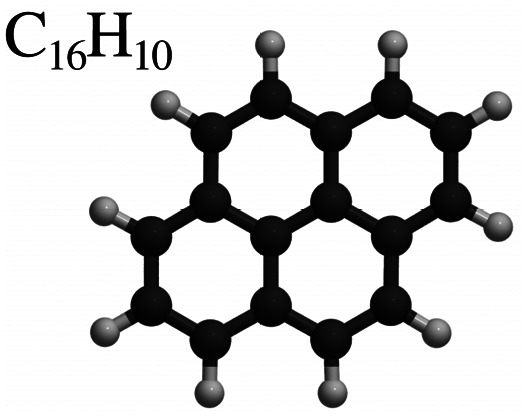
Pyrene	C_16_H_10_	
Pentacene	C_22_H_14_	
Large organic molecules		
Decacyclene	C_36_H_18_	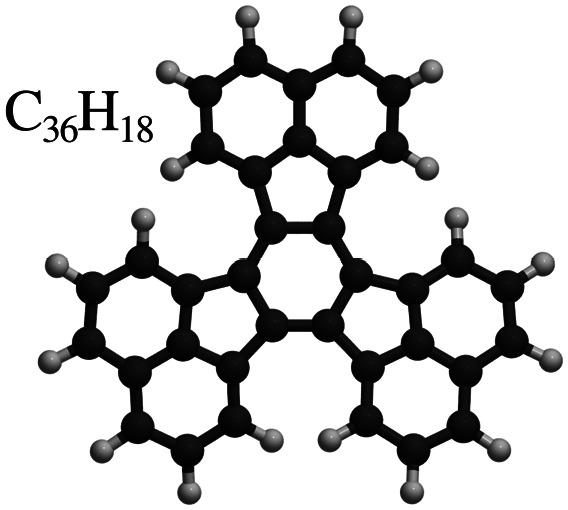
Triphenyl compounds	P(C_6_H_5_)_3_	
Metal-phthalocyanines	C_32_H_16_CoN_8_	
Tetrapyridylporphyrin	C_40_H_26_N_8_	

After benzene (C_6_H_6_), the prototypical aromatic molecule, we will discuss heterocyclic planar rings such as pyrazine (C_4_H_4_N_2_) and s-triazine (C_3_H_3_N_3_), as well as five-membered rings such as pyrrole (C_4_H_5_N) and cyclopentadienyl (C_5_H_5_). We further include polycyclic aromatic hydrocarbons (PAHs) such as naphthalene, pyrene (C_16_H_10_), and pentacene (C_22_H_14_). Among the various π-conjugated molecules relevant to organic electronics, C_22_H_14_ stands out as a well-characterised reference system, widely recognised as a model compound for organic semiconductors and thin film growth.^10,56–59^ For completeness, we also briefly touch upon more structurally complex adsorbates such as porphyrins and phthalocyanines, although these lie beyond the primary scope of this review.

In what follows, we first provide the physical background of surface diffusion and outline key experimental techniques for measuring nanoscale motion. Subsequently, we discuss recent results on the dynamics of these molecular systems across various substrates, including metals and 2D materials.

## Background on diffusion and measurements

### Surface diffusion and energy barriers

Surface diffusion describes the thermally activated motion of adatoms, molecules, or clusters across a material surface. At finite temperatures, diffusing species undergo continuous thermal motion, and their trajectories can be monitored in simulations or experiments, which is referred to as tracer diffusion.^9,60,61^ A classical model typically considers motion in a one-dimensional (1D) periodic potential (Fig. 5(b)). At low surface temperatures (*k*_B_*T* ≪ *E*_b_), adsorbates remain mostly confined to the minima of the adsorption potential, occasionally acquiring enough energy to hop to adjacent sites. In this regime, the hopping rate *ϒ* is described by an Arrhenius-type law:^20,60–62^1
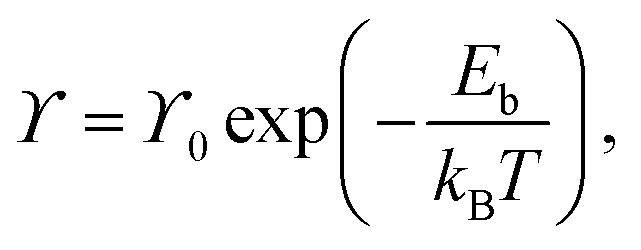
where *ϒ*_0_ is the attempt frequency, often approximated by the frequency of the frustrated translational mode (T-mode) at the bottom of the potential well.^60,61^ The diffusion coefficient *D* associated with this hopping process also follows Arrhenius behaviour:2
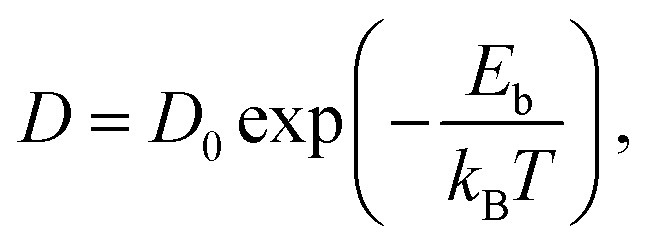
with the prefactor 
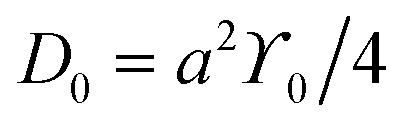
 for isotropic two-dimensional diffusion on a square lattice of jump length *a*. This traditional picture of surface diffusion envisions a random walk in which the adatom hops over energy barriers from one favourable adsorption site to another, along the energetically most favourable route of the potential energy surface (PES).^8,61,62^

Eqn (1) and (2) hold regardless of whether diffusion is measured in real space or reciprocal space as described below. Thus, if measurements as a function of temperature under otherwise constant conditions are plotted in an Arrhenius representation, an activation energy *E*_a_ can be directly extracted. It should be noted that the activation energy *E*_a_ extracted from Arrhenius plots does not necessarily equal the true adiabatic energy barrier *E*_b_ of the potential energy surface.^61^ Nevertheless, this effective barrier remains a practical and widely used approximation for interpreting experimental data.

Rate theories such as transition state theory (TST) estimate *ϒ* using thermodynamic arguments, where the hopping rate is proportional to the ratio of the partition functions in the transition state, *Z*_s_ and the well state, *Z*_0_, *i.e.*
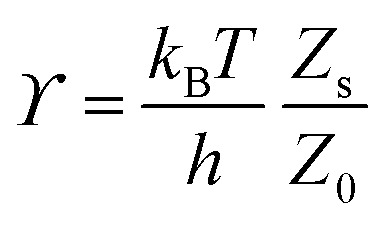
.^60,61^ In traditional TST, the concept is quite simple, and the hopping rate is related to the rate of passage of the adsorbate through the transition state at the top of the energy barrier. Assuming a simple harmonic oscillator potential 
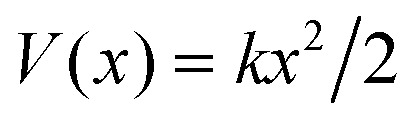
, the attempt frequency becomes *ϒ*_0_ = *ω*/2π. However, classical models neglect essential effects. For instance, they assume a free-atom kinetic energy at the transition state and ignore quantum tunnelling, which may be significant for light particles.^63^ More critically, they omit energy exchange between the adsorbate and the substrate, which is central to more sophisticated treatments such as Langevin or Fokker–Planck equations.^61,64^

**Fig. 1:**
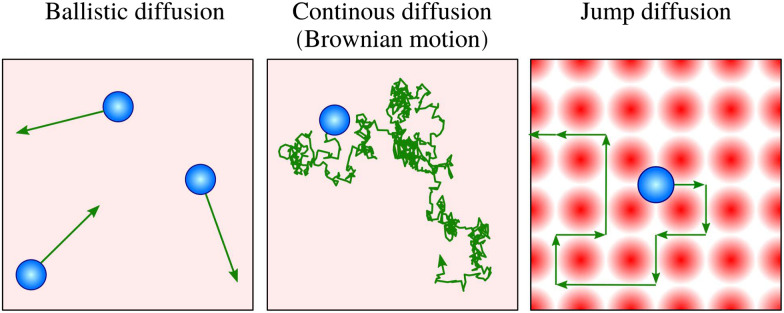
Three simple modes of surface diffusion with schematic trajectories shown as green lines, with the blue circles illustrating the moving adsorbates. Ballistic or 2D gas-like motion means that the adsorbates move in a linear fashion. The simplest form of 2D diffusion is continuous random motion, also known as Brownian motion in other fields, such as particles in a liquid. In the case of jump diffusion, the adsorbates move between vacant sites of the underlying surface (illustrated by the red circles) where the potential energy is smallest.

This simple hopping picture captures essential features of surface diffusion and holds at low temperature for a number of systems, specifically for self-diffusion of single atoms across transition metal surfaces.^8,62^ However, diffusion will become more complex for larger molecules as well as with increasing temperature and more importantly, it cannot describe dynamics on weakly interacting surfaces. In contrast to strongly bound systems, aromatics such as benzene interact weakly with inert surfaces like graphite. In such cases, the low adsorption energy gives rise to alternative diffuse regimes such as ballistic or Brownian motion as shown in Fig. 1, which requires to introduce the concept of atomic-scale friction to describe the full range of molecular motion.

### Atomic-scale friction

Atomic-scale friction refers to energy dissipation between a diffusing adsorbate and the substrate, which influences the rate and nature of surface diffusion. In surface diffusion, energy dissipation is captured by a friction coefficient *η*. Within the Langevin description of dynamics, which provides a classical treatment of diffusion and vibrational motion, energy dissipation is introduced *via* a friction coefficient *η*, while the PES and thermal energy (*k*_B_*T*) define the topography over which motion occurs. The Langevin equation for the motion of an adsorbate *j* with mass *m* at position **R**_*j*_ on a two-dimensional PES *V*(**R**) is given by:^9,60,61^3
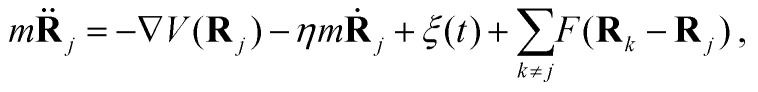
where *η* represents the friction in terms of energy loss to the substrate, *ξ*(*t*) is a stochastic force describing thermal fluctuations, and the final term includes interactions with other adsorbates.^60,65^ This model integrates vibrational and translational motion, treating them on equal footing, and is well-suited for systems where quantum effects are negligible. While, historically, surface diffusion and adsorbate vibrations were considered separately due to their different timescales, it is now recognised that they are intimately linked.^61^ For example, the pre-exponential factor *ϒ*_0_ in transition-state theory (TST) often corresponds to the frustrated translational mode frequency at the bottom of the potential well. In more complex systems, molecular diffusion may involve the excitation of internal degrees of freedom, which can also be captured in Langevin-based models.^66^

**Fig. 2:**
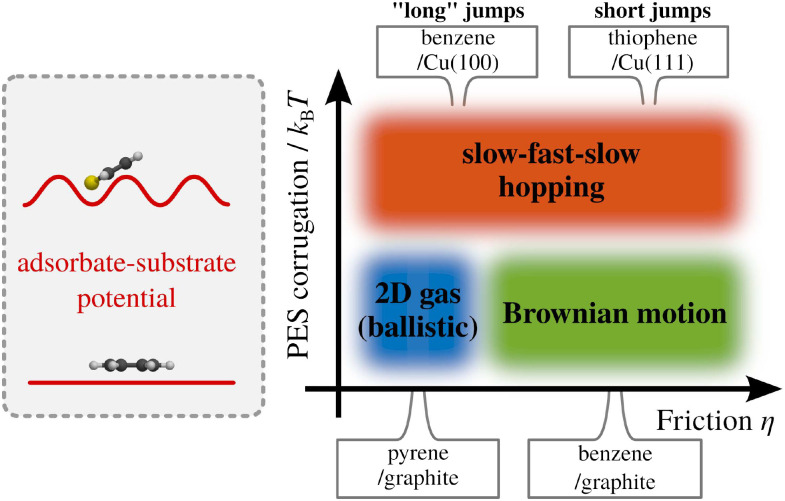
Schematic drawing illustrating the three simple models of surface diffusion, with example systems. The motion depends on the energy dissipation rate (friction *η*) and the potential energy surface (PES) corrugation relative to *k*_B_*T*. For a negligible corrugation of the PES (*i.e.* a more or less “flat” adsorbate-substrate PES as illustrated on the left), and simultaneously a low friction *η*, ballistic motion is expected to occur (*e.g.* pyrene on graphite^67^). Keeping the corrugation of the PES low while increasing *η* gives rise to continuous random (Brownian) motion (*e.g.* benzene on graphite^68^). If the corrugation of the PES becomes significant with respect to *k*_B_*T*, the diffusive motion tends to follow the periodicity of the underlying PES, giving rise to hopping motion. In the low-friction regime, long jumps may occur (*e.g.* ref. 69 and 70), whereas high *η* results in short jumps (*e.g.* ref. 66 and 71).

Within the Langevin description of dynamics, the two variables which largely determine the type of motion, as illustrated in Fig. 1, are the atomic-scale friction *η* and the diffusion barrier *E*_b_ or, in other words, the corrugation of the PES relative to the thermal energy *k*_B_*T*. Fig. 2 provides an illustration of these relationships and the regimes where ballistic, Brownian, and jump diffusion dominate, with corresponding examples.

• Ballistic motion: for a corrugation of the PES that approaches zero compared to the energy of the diffusing adsorbates and a negligible coupling to the substrate, one expects to observe so-called ballistic motion on sufficiently small length and timescales. Ballistic or 2D gas-like motion means that the adsorbates move in a linear fashion over the surface (Fig. 1), as *e.g.* observed for pyrene on graphite.^67^

• Brownian motion: as seen for a weak PES corrugation but increased friction, the simplest form of 2D diffusion is expected to occur: continuous random motion, also known as Brownian motion in other fields, such as particles in a liquid and *e.g.* observed for benzene on graphite.^68^

• Hopping (jump) diffusion: for sufficiently low temperatures or large diffusion barriers, the atomic scale motion becomes dominated by the periodic arrangement of the surface atoms, and the motion turns into discrete hops or jumps between preferred adsorption sites.^20,62^

From a theoretical viewpoint, friction *η* not only governs energy dissipation but also affects the hopping rate when activated diffusion occurs as described in Kramers' turnover theory:^64,72^

• In the low-friction regime, the particle infrequently gains sufficient energy to cross the diffusion barrier. Once it does, the lack of rapid energy loss promotes multiple or long jumps.

• In the high-friction regime, although energy is readily gained, strong dissipation increases the likelihood of barrier recrossing, limiting motion to single jumps.

Therefore, TST overestimates the rates and is only expected to provide an upper limit of the hopping rate.^9,61^

As further described below in the section Motion in reciprocal space, characteristic signatures of each motion regime manifest distinctly in experimental observables. However, real systems rarely conform to idealised diffusion models; for instance, actual jumps do not occur instantaneously, as implicitly assumed in eqn (5). While analytic models provide essential qualitative insight into underlying mechanisms, they often fall short in quantitatively reproducing experimental data.^61^ Therefore, a more comprehensive understanding typically requires integration with computational approaches such as molecular dynamics or kinetic Monte Carlo simulations.^67,69,73,74^

### Scanning probe microscopy

**Fig. 3:**
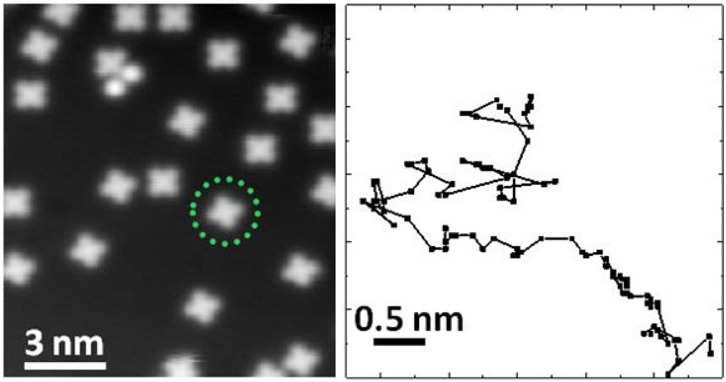
Cobalt Phthalocyanine (C_32_H_16_CoN_8_) diffusion on Ag(100), illustrating the use of STM for tracking molecular motion. The left panel shows an STM snapshot at *T* = 49 K, while the right panel depicts the centre-of-mass trajectory of a single molecule as marked with a green circle on the left, tracked over a total of ≈4.5 hours with a resulting mean-square displacement of 20 Å^2^. (Reprinted with permission from ref. 18, Copyright 2015 by the American Chemical Society).

The first observations of single-atom diffusion were made using field emission and field ionisation microscopy (FIM), which employed an image-anneal-image methodology. However, these early studies were restricted to specific systems and geometries.^8^ With the advent of scanning tunnelling microscopy (STM), real-space investigation of surface diffusion experienced a major breakthrough. STM offers atomic-scale resolution across a wide variety of materials and enables *in situ* imaging above cryogenic temperatures, the so-called image-while-hot approach. In this context, STM has become a powerful technique for investigating thermally activated processes such as surface diffusion. By correlating successive static images into a time series, so-called “video STM” allows for a direct visualisation of adsorbate dynamics (Fig. 3). More advanced protocols, including those developed by Hahne *et al.*, extract residence times of atoms beneath the STM tip from temporal data series,^75^ and have also been employed for organic molecules by measuring the diffusive noise, as demonstrated by Ikonomov *et al*.^76^

Several instrumental STM studies have explored the diffusion of larger organic molecules adsorbed on metal surfaces as shortly described in the section Other and more complex organic molecules, where the diffusion events are sufficiently slow to be resolved within STM scan times.^77–82^ For instance, Weckesser *et al.* investigated the 1D diffusion of 4-*trans*-2-(pyrid-4-yl-vinyl)benzoic acid (PVBA) on Pd(110), determining activation energies from temperature-dependent measurements.^77^ Likewise, Loske *et al.* extracted the diffusion barrier for C_60_ using atomic force microscopy (AFM) in conjunction with island nucleation theory.^83^ While STM allows measurement of the diffusivity, activation energy *E*_a_, and attempt frequency, its applicability is limited by the time-resolution of the scanning process. This makes it difficult to probe the fast motion of small molecules such as CO^84,85^ or water,^20,86^ and also the surface mobility of dimers and trimers remains challenging.^18,84,85^ Similarly, aromatics such as benzene moving on weakly interacting surfaces like graphite are difficult to capture in STM measurements.^10^

### Quasielastic scattering and surface dynamics

Quasielastic neutron scattering (QENS) is a powerful technique for probing molecular diffusion through the detection of Doppler broadening in the energy distribution of scattered neutrons. The term “quasielastic” refers to small energy changes near the elastic peak, as opposed to inelastic scattering that would involve phonon excitation. Techniques such as neutron time-of-flight (TOF) and neutron spin-echo (NSE) spectroscopy offer the required energy and time resolution to investigate diffusive dynamics at interfaces.^20,87^ Using porous media such as exfoliated graphite, which provide a large effective surface area and facilitating in-plane scattering geometry, allows QENS to probe adsorbate dynamics on solid surfaces. Particularly for organic molecules on graphite, QENS benefits from a strong contrast between the scattering cross-sections of hydrogen and carbon,^87,88^ enabling distinction between the dynamics of the adsorbed layer and that of the substrate.

NSE measurements provide direct access to the intermediate scattering function (ISF) *I*(*Q*,*t*) as a function of in-plane momentum transfer *Q* = |**Q**| = |**K**_**f**_ − **K**_**i**_|, enabling time-resolved characterisation of surface dynamics, as described in more detail below. In contrast, neutron TOF spectroscopy yields the scattering function SF *S*(*Q*,Δ*E*) by converting the time-of-flight spectra into energy-resolved scattering data.^89^ The scattering function *S*(*Q*,Δ*E*), also known as the dynamic structure factor, is the temporal Fourier transform of the ISF and captures the spectral distribution of energy exchanges due to molecular motion.

### Helium spin-echo

**Fig. 4:**
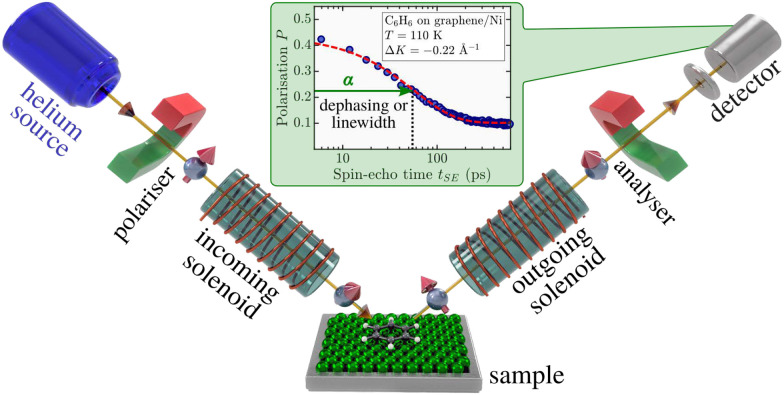
Schematic of a helium spin-echo (HeSE) apparatus. The spin of the ^3^He atoms acts as an internal timer that can be manipulated by magnetic solenoids before and after scattering. The interference of two time-separated wave packets allows detection of surface motion *via* a change in spin-polarisation upon scattering from the moving adsorbates. The inset shows a typical intermediate scattering function (ISF, *I*(Δ*K*,*t*_SE_)), reflecting the time-dependent correlation of the adsorbate dynamics *via* measurement of the quasi-elastic linewidth or dephasing rate *α*.

Helium spin-echo (HeSE) spectroscopy – also referred to as quasielastic helium atom scattering (QHAS) – extends the neutron spin-echo principle to neutral helium atoms. It is uniquely suited for investigating single-particle dynamics at surfaces due to its high surface sensitivity and exceptional energy resolution:^61^ HeSE probes molecular diffusion by detecting Doppler-induced energy changes caused by surface motion, manifested as a polarisation decay of the scattered beam (see inset of Fig. 4). The challenge of limited monochromaticity in supersonic He beams is overcome by encoding the energy exchange into the nuclear spin of ^3^He atoms, adopting concepts from neutron spin-echo techniques.^87^

As shown in Fig. 4, a spin-polarised ^3^He beam is split into two coherent wave packets that arrive at the surface at different times separated by *t*_SE_ – the spin-echo time. After interaction with the surface, the wave packets are recombined and their interference pattern is measured *via* the resulting beam polarisation. Energy changes due to surface motion alter the relative phase between the wave packets, leading to a measurable depolarisation. This technique effectively uses the ^3^He nuclear spin as an internal “timer”. Due to the low kinetic energy of He atoms (<10 meV), the surface is not perturbed, while the large scattering cross-section of individual adsorbates ensures high sensitivity.^20,90,91^

A typical HeSE measurement yields the polarisation as a function of spin-echo time *t*_SE_ at a fixed surface-parallel momentum transfer Δ*K* = |Δ**K**|. This polarisation is directly proportional to the intermediate scattering function *I*(Δ*K*,*t* = *t*_SE_), the temporal Fourier transform of the van Hove pair correlation function.^61,92^*I*(Δ*K*,*t*) has been calculated analytically for various prototypical types of surface motion as described below.

### Surface diffusion measurements

In summary, STM and reciprocal space techniques (QENS & QHAS) are complementary: while STM offers atomically resolved real-space images typically at lower temperatures, reciprocal space methods provide access to dynamic processes at shorter timescales and elevated temperatures.^93^ The fundamental difference between real-space and reciprocal-space techniques lies in how spatial and temporal averaging is performed. In real-space techniques like STM, temporal averaging occurs over long trajectories or scan times, providing snapshots of molecular positions. While STM enables direct imaging of the visited sites, as shown in Fig. 3, it does not allow one to obtain detailed information about the transition between those sites or the path of motion. Reciprocal-space techniques, such as scattering experiments, perform spatial and temporal averaging over the entire ensemble, but maintain access to the detailed dynamics of the motion. Although the data are indirect and are more difficult to analyse than their real space counterparts, they convey the full breadth of microscopic detail. Consequently, while real-space techniques offer intuitive visualisation, reciprocal-space methods convey the entire picture of surface motion, especially on shorter time scales.^20,61^

### Motion in reciprocal space

**Fig. 5:**
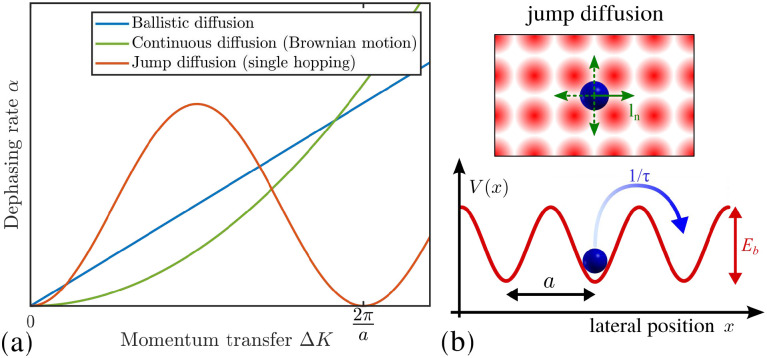
(a) The dephasing rate *α* shows a linear/quadratic/periodic dependence upon momentum transfer Δ**K** for ballistic/Brownian/hopping motion. Ballistic diffusion gives a Gaussian decay in *I*(Δ**K**,*t*), with *α* varying linearly with Δ**K**. Random (Brownian) motion gives an exponential decay in *I*(Δ**K**,*t*) with *α* varying quadratically with Δ**K**. For hopping motion, the *α*(Δ*K*) dependence is sinusoidal with a period 2π/*a* given by the jump length *a*. (b) Illustration of adsorbate jumps (blue sphere) on a square lattice in top and side view. Hops to neighbouring sites are described by jump vectors ***l***_*n*_ and probabilities *p*_*n*_. In the simplest case with nearest-neighbour jumps of spacing *a*, *α* as plotted in (a) follows the periodicity in reciprocal space according to eqn (5).

An important question is how one can differentiate between different types of diffusive regimes on a surface based on reciprocal space measurements. The signatures of different diffusive regimes are contained in the dependence of the dephasing rate *α*(Δ**K**) of the ISF or the quasi-elastic broadening *Γ*(Δ**K**) of the SF on the momentum transfer Δ**K**.^20,61,89,94^ The three simple model mechanisms, Brownian, ballistic and hopping motion, and their signatures form references for the interpretation of QHAS and QENS experiments, provide a more general insight into the underlying mechanism of surface diffusion. Following the behaviour in real space based on the van Hove pair correlation function *G*_s_(**R**,*t*), the different diffusive modes (see the different trajectories **R**_*j*_ in Fig. 1) correspond to specific signatures in reciprocal space. At low coverages (*i.e.* where adsorbate interactions can be neglected) it corresponds to a specific “fingerprint” for different self-diffusive regimes as shown in Fig. 5(a).

#### Ballistic

Ballistic or 2D gas-like motion means that the adsorbates move in a linear fashion over the surface (Fig. 1). Note that on small enough length and timescales, all motion appears Ballistic while at low enough adsorbate densities, Brownian dynamics can transform into ballistic motion, *i.e.*, molecular collisions become negligible and molecules move in a linear fashion.^67^

#### Brownian motion

The typical signature of continuous random (Brownian) motion is the quadratic dependence of *α* (or *Γ*) upon the momentum transfer Δ**K***via α*(Δ**K**) = *D*Δ**K**^2^ as illustrated in Fig. 5(a). For sufficiently small Δ**K**, or in the limit of large length scales in real space, all diffusive motion must conform to this macroscopic limit.^61^ Another aspect of Brownian motion is that the curvature of the quadratic dependence of *α*(Δ**K**) corresponds directly to the diffusion coefficient *D*, and in fact, it can be used to determine *D* from an experimental data set.^68^ Finally, in the case of Brownian motion, the diffusion coefficient is directly related to the atomic-scale friction *η via* Einstein's relation:^61,68^4
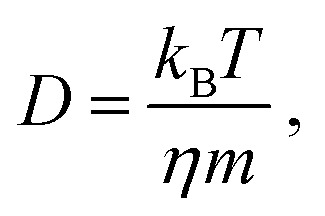
where *m* is the mass of the diffusing adsorbate.

#### Jump diffusion

Based on the analysis of neutron scattering data from 3D liquids, an analytic model that describes hopping motion was first introduced by Chudley & Elliot.^95^ Following that work, similar expressions were later developed to describe the hopping of adsorbates on surfaces, which is usually referred to as the Chudley–Elliott (CE) model.^9,60,61,96^ It assumes that an adsorbate instantaneously jumps from one adsorption site to the other, with the probability *p*_*n*_ = 1/*τ*_*n*_ (Fig. 5(b)). The dephasing rate *α*(Δ**K**) exhibits then the typical functional dependence in terms of Δ**K**:^61,69^5
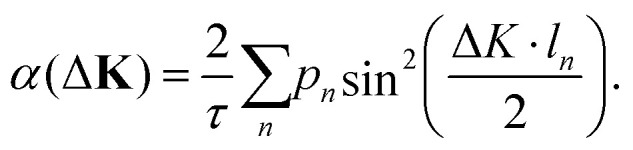


Based on the CE model (5), the dephasing rate *α* follows a sinusoidal dependence (sin^2^) *versus* momentum transfer Δ**K** as shown in Fig. 5(a). The amplitude of this sinusoidal shape according to (5) is given by 1/*τ*, with *τ* being the mean residence time between motion from one adsorption site to the other.

As illustrated in Fig. 5, the dephasing rate *α*(Δ**K**) then follows the periodicity of the lattice in real space, giving rise to a typical sin^2^ dependence *versus* Δ**K** according to (5): For any momentum transfer Δ**K** that corresponds to multiples of the lattice spacing in real space (2π/*a*), the ISF remains constant as a function of time *t*, while in between it decays quickly. The position where the ISF remains constant and where (5) becomes a minimum in terms of Δ**K** corresponds to the Bragg diffraction peaks for the surface (at a Bragg peak, the lattice sites scatter in phase and are insensitive to jumps between sites, resulting in a dephasing rate, *α* = 0).

When a number of different jump lengths ***l***_*n*_ in (5) are possible, these will simply contribute to the overall value of *α* as a number of Fourier components, while the minima of *α*(Δ**K**) will still be at Bragg peak positions of the substrate lattice. The CE model contains also Brownian diffusion as a long range diffusion limit, *i.e.* for Δ*K* → 0 the broadening converges to a parabola^73^ and thus approaches the same Δ**K** dependence as for Brownian motion. Finally, the diffusion coefficient *D* for 2D motion along a particular surface direction (given by Δ**K**) can then be calculated from the hopping rate as determined from the CE model *via D* = 1/4〈*l*〉^2^*ϒ*, where *ϒ* is the hopping rate and 〈*l*〉 is the mean jump length.^9,61,69^

## Benzene on flat metals and graphite

Benzene (C_6_H_6_) is the simplest aromatic molecule, possessing a planar, highly symmetric π-electron system, rendering it an exemplar model for the study of π-surface interactions. Its chemical stability and structural rigidity facilitate physisorption without molecular fragmentation on most metal surfaces, making it particularly suitable for fundamental studies in surface science. Typically, benzene adsorption is conducted on atomically flat substrates, such as Cu(111), graphite, or graphene, which are well-characterised and offer a controlled environment to isolate and examine molecule-surface interactions with precision.^11,28,58,67,97–99^ Furthermore, benzene serves as a prototype for more complex polycyclic aromatic hydrocarbons (PAHs), functionalised aromatics, and organic semiconductors, providing a foundational framework for understanding their interactions with surfaces.

**Fig. 6:**
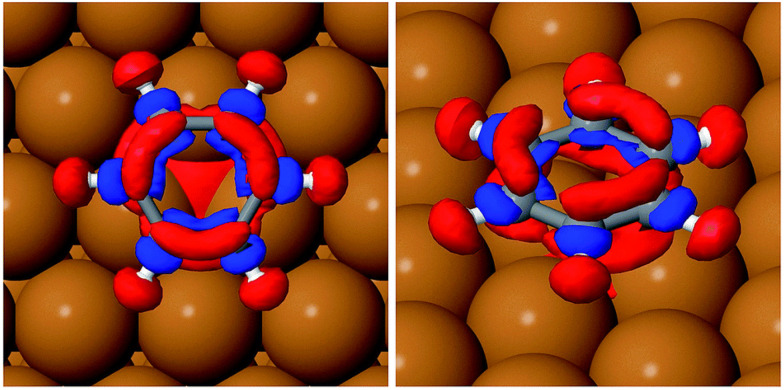
Top view (left) and side view (right) of the charge density difference plots illustrating charge transfer upon adsorption of C_6_H_6_ on the Cu(111) hcp site with C_6_H_6_ rotated by 30° with respect to 
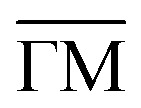
-azimuth. Red contours represent an electron density increase of 0.005 e Å^−3^, while blue contours indicate an electron density decrease of 0.005 e Å^−3^. The configuration corresponds to the most stable adsorption site with an adsorption energy of −1.050 eV as computed using the PBE functional combined with the vdW correction *via* the Tkatchenko–Scheffler scheme. (Reprinted from ref. 97 under the terms of the Creative Commons CC BY license).

**Fig. 7:**
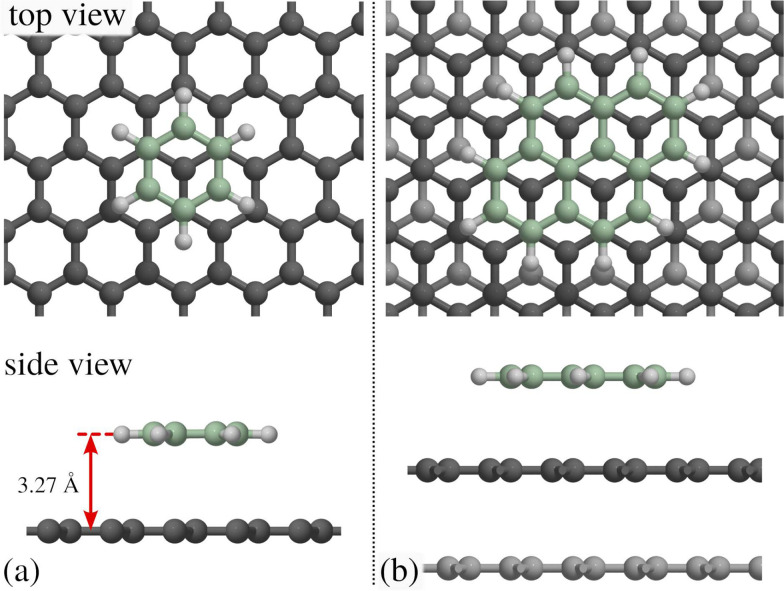
(a) Top and side view of benzene (C_6_H_6_) adsorbed on graphene, giving rise to the typical planar face-to-face adsorption geometry with an adsorption height of ≈3.3 Å. (b) Similarly, pyrene (C_16_H_10_) adsorbed on graphite adopts the AB stacking of the substrate. The carbon atoms of the aromatics are shown in light green for illustrative purposes.

In summary, the investigation of benzene adsorption on metal and graphitic surfaces provides a robust, controlled, and theoretically tractable platform for exploring the nature of molecule-surface interactions, diffusion mechanisms, and modifications to electronic structure.^26,28,100–102^ This platform enables researchers to probe several critical phenomena. Firstly, adsorption studies illuminate the delicate balance between vdW forces, π-d hybridisation (particularly on metal surfaces), π–π interactions on graphene and graphite and the distinction between physisorption and chemisorption.^103^ These interactions dictate the strength and nature of the molecule–surface bond, influencing subsequent chemical and physical processes. The left part of Table 1 provides an overview of C_6_H_6_ adsorption energies *E*_ads_ using DFT with vdW corrections and experimentally obtained desorption energies *E*_des_ for Cu and graphite. As further illustrated in Fig. 6 and 7(a), benzene (C_6_H_6_) in particular is typically characterised by a flat adsorption geometry on both graphene and flat metal surfaces, at least in the sub-monolayer regime.^101,104^ Secondly, understanding the mobility of benzene on surfaces – whether through activated hopping or Brownian motion – offers valuable insights into the energy landscapes and surface friction at the atomic scale, which are pivotal for applications in chemical vapour deposition, nanotechnology and tribology.^6,7,33,55,105–107^ Thirdly, the adsorption process induces notable changes in electronic structure, which can be investigated using techniques such as photoemission spectroscopy or DFT. These studies reveal critical phenomena, including charge transfer (see Fig. 6 for C_6_H_6_ on Cu(111)), shifts in work function, and orbital hybridisation, all of which are essential for tailoring surface properties in electronic and catalytic applications.^46–48^

Table 1 also illustrates how benzene adsorption systems, particularly on metal surfaces, are widely used to validate and refine computational methodologies due to their well-characterised interaction profiles,^26–30^ by summarising the theoretically obtained adsorption and the experimental desorption energies of a few representative benzene systems. Therefore, extensive theoretical investigations have probed the interplay of electronic structure, vdW interactions, and surface geometry,^28,30,98,101,108^ and several studies have evaluated the performance of various exchange–correlation functionals and dispersion correction schemes.^101,109–115^ Collectively, these efforts establish benzene adsorption as a prototypical system for benchmarking theoretical approaches in surface science.

**Fig. 8:**
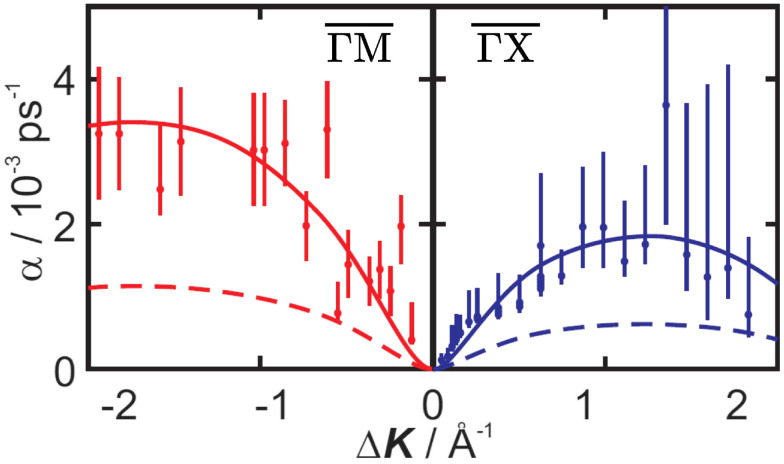
Diffusion of 0.07 ML Benzene (C_6_H_6_) on Cu(100) at 170 K follows jump diffusion. The dephasing rate *α* obtained from HeSE measurements (red and blue dots) follows the periodicity of the underlying Cu(100) substrate, as is the case for jumps between adjacent adsorption sites. Dashed and solid lines represent MD simulation results for translational jumps only and for translational jumps plus rotation, respectively. Rotational contributions, as shown by the solid lines, clearly increase dephasing, with both motions being associated with a friction coefficient of *η* = 0.4 ps^−1^. (Reprinted with permission from ref. 70, Copyright 2016 by the American Chemical Society).

Beyond its role in computational benchmarking, benzene adsorption and its nanoscale dynamics provide a fundamental reference point for studying larger π-conjugated systems. It enables systematic exploration of substituted aromatics, molecular electronics, and self-assembled monolayers, which are central to applications in sensing, organic electronics, and surface functionalisation.^31–33^

### Nanoscale motion and dynamics of benzene

The right part of Table 2 provides a summary of studies investigating benzene diffusion and nanoscale motion on flat metal surfaces and graphite. It clearly shows that the variation in interaction strength between benzene and different metal surfaces, as well as between C_6_H_6_ and graphite, gives rise to distinctly different types of motion. In particular, the weak binding of C_6_H_6_ to graphite^117,119^ leads to low diffusion barriers, making experimental studies of single-molecule diffusion processes challenging. In the following, we describe in more detail the molecular motion of C_6_H_6_ on Cu(100),^70^ Cu(111),^97^ and graphite,^68,118^ together with theoretical approaches to understand benzene motion on surfaces.^120,121^

**Table 2:** Summary of adsorption, diffusion, and friction parameters for benzene (C_6_H_6_) on different surfaces. In the left part, C_6_H_6_ adsorption (*E*_ads_) and desorption (*E*_des_) energies with the corresponding references are summarised. Desorption energies are experimental values obtained from thermal desorption spectroscopy, where available; adsorption energies were computed using DFT with vdW correction according to the Tkatchenko–Scheffler scheme. The right part summarises diffusion characteristics with the corresponding methods and references

Surface	Ad- & desorption energy *E*_ads_/*E*_des_ (eV)		Ref.	Motion	Activation energy *E*_a_ (meV)	Friction	Methods	Ref.
Cu(100)	−1.47	—	70	Jump diffusion	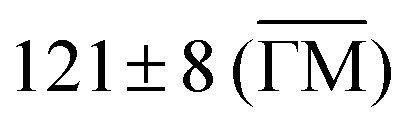	0.4 ps^−1^	HeSE, DFT & MD	70
					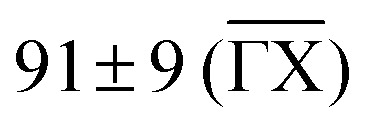			
Cu(111)	−1.0	0.58	97, 101, 104 and 116	Jump diffusion	35 ± 1	—	HeSE & DFT	97
Graphite	−0.40	0.50	67, 103 and 117	Brownian	17 ± 12	2.2 ps^−1^ (0.5 ML)	HeSE, NSE & MD	68
				Brownian with inter-molecular friction		0.50 ps^−1^ (0.1 ML)	Neutron TOF	118
						1.82 ps^−1^ (1.0 ML)	Neutron TOF	

#### Benzene on Cu surfaces

As can be seen in the overview Table 2, the diffusion of benzene on Cu(100) is a “classic” example of activated jump diffusion due to the comparably large corrugation of the PES, giving rise to a site-to-site hopping motion. Hedgeland *et al.*^70^ illustrated that benzene molecules preferentially adsorb at fourfold hollow sites and overcome diffusion barriers located at the bridge positions. As shown in Fig. 8, HeSE measurements of temperature-dependent dephasing rates for 0.07 monolayer (ML) C_6_H_6_ reveal anisotropic diffusion characterised by effective activation energies of 121 ± 8 meV along the 
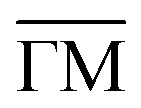
 direction and 91 ± 9 meV along 
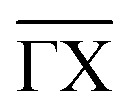
.

Complementary DFT calculations, including vdW corrections, provide an energy hierarchy of adsorption sites: hollow > bridge > top. These calculations yield energy barriers of 351 meV over the bridge site and 500 meV over the top site. To match the experimentally observed diffusion dynamics, Langevin simulations were employed, resulting in adjusted, effective energy barriers of 122 meV and 172 meV for bridge and top sites, respectively.

Beyond these energetic considerations, MD simulations within a Langevin framework, as introduced in the section Atomic-scale friction, provide a best-fit value of *η* = 0.4 ps^−1^ by comparing simulation results to the experimentally measured dephasing rates *α*. According to Kramers’ theory, the diffusion rate's dependence on friction is non-monotonic and reflects the complex interplay between thermal noise and dissipation in activated surface transport. Crucially, the observed diffusion rate exceeds that predicted by conventional point-particle models by a factor of 3.0 ± 0.1, pointing to the significance of internal molecular degrees of freedom. Rotational motion with the same friction coefficient *η*, was identified as a key contributor that facilitates translational motion by effectively lowering energy barriers and enabling alternative diffusion pathways.^70^ At low momentum transfers (<0.5 Å^−1^), HeSE measurements further show a local maximum in the dephasing signal, indicating repulsive interactions between adsorbates and deviations from the simple CE diffusion model. These findings highlight the need to treat the adsorbate as an extended object with additional dynamic variables to accurately describe nanoscale motion on surfaces.

In contrast to the highly corrugated Cu(100) surface, the Cu(111) surface presents a smoother potential energy landscape, with the adsorption sites forming a Bravais lattice. HeSE measurements presented by Sacchi *et al.*^97^ indicate that benzene undergoes activated jump diffusion between these sites. The nature of the adsorption sites has been investigated through both experiment and theory. While the experimental polarisation data show no evidence of a second exponential decay – excluding bridge or degenerate hollow site adsorption – they remain consistent with adsorption on top or non-degenerate hollow sites. DFT calculations with vdW corrections identify hollow sites as global minima and top sites as shallow local minima, indicating diffusion likely occurs *via* jumps between hollow sites, bypassing top sites due to their higher energy.

Moreover, HeSE measurements yield an effective activation energy of 35 ± 1 meV for benzene diffusion on Cu(111), approximately one-third of the barrier on Cu(100), reflecting the lower corrugation and higher symmetry of Cu(111). DFT calculations employing the transition state (TS) and transition state scaling correction scheme (TSSCS) methods yield diffusion barriers of 21 meV and 18 meV, respectively, both within chemical accuracy. Analysis identifies the HCP hollow site as the most stable adsorption site, with FCC hollow sites lying 10–14 meV higher in energy. The diffusion barrier between the HCP and bridge sites is approximately 20 meV, consistent with experimental measurements. Rotational barriers around the *C*_6_ symmetry axis range from 12–23 meV. The rate-limiting step in the diffusion pathway depends on the computational method: from HCP-Rotated (HCP-R) to Bridge-Rotated (BR-R) in the TS and TSSCS schemes; from HCP-R to FCC-R in the G06 method; and from HCP-Inclined (HCP-I) to Bridge-Inclined (BR-I) in the Ortmann–Bechstedt–Schmidt (OBS) method. Nonetheless, all approaches agree that diffusion occurs *via* low-barrier transitions between non-equivalent hollow sites, contrasting the more corrugated Cu(100) landscape.^97^

#### Benzene on graphite

The first experimental measurement of benzene diffusion, as summarised in Table 1, was reported by Hedgeland *et al.*^68^ for C_6_H_6_ on graphite. The study demonstrated that C_6_H_6_ on graphite exhibits atomic-scale, continuous Brownian motion, representing a regime of free diffusion on a flat energy landscape. This type of motion was notably distinct from earlier studies, which predominantly observed activated hopping diffusion. Diffusion data were obtained for 0.5 ML benzene on graphite at 140 K using both HeSE for C_6_H_6_ on highly oriented pyrolytic graphite (HOPG) and NSE measurement for C_6_H_6_ on exfoliated graphite.^68^

From a theoretical perspective, Brownian motion corresponds to a purely diffusive regime with negligible surface corrugation. The 2 experimentally observable scattering functions, the ISF and the SF, can both be derived from the self-correlation function *G*_s_(***R***,*t*), with the ISF exhibiting a purely exponential decay. Notably, the dephasing rate *α* of the ISF and the full width at half maximum (FWHM) *Γ* of the SF follow the relations:

6 *Γ*(Δ**K**) = 2*ħα*(Δ**K**) = *2ħD*Δ**K**^2^.

**Fig. 9:**
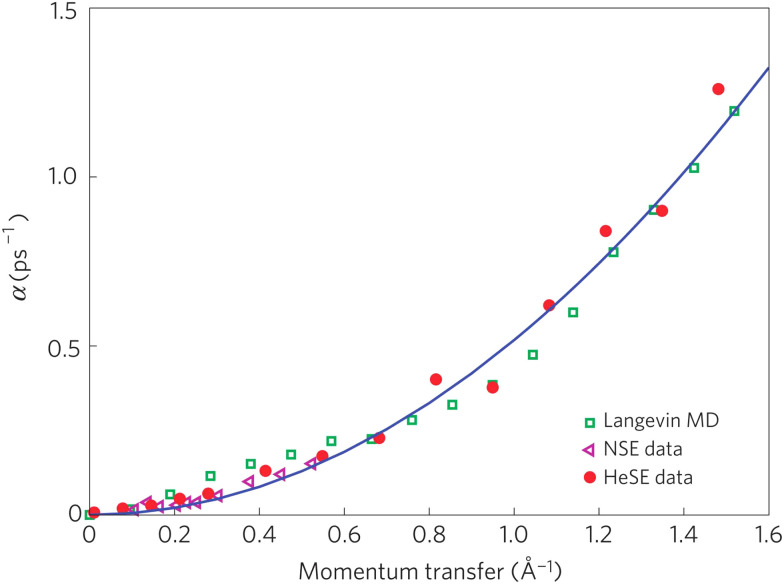
Diffusion of 0.5 ML Benzene (C_6_H_6_) on graphite at 140 K follows Brownian motion. The dephasing rate *α* obtained from HeSE measurements (circles), NSE measurements (triangles), and Langevin molecular dynamics (MD) simulations (squares) closely follows the parabolic fit (dark blue line), confirming the theoretical prediction of Brownian motion. (Reprinted with permission from ref. 68, Copyright 2009 by Springer Nature).

Indeed, as shown in Fig. 9, the dephasing rate α increases parabolically with momentum transfer Δ**K** according to eqn (6), which is characteristic of Brownian motion in a viscous regime with high kinetic friction. An Arrhenius analysis of the dephasing rate yielded an exceptionally low activation energy of 17 ± 12 meV, confirming that the surface corrugation is minimal and thermal motion is sufficient to drive diffusion without significant activation. The diffusion coefficient was determined to be *D* = (5.39 ± 0.13)⋅ 10^−9^ m^2^ s^−1^ at 140 K.^68^ Although specific adsorption sites are not detailed in the study, previous investigations have identified a (
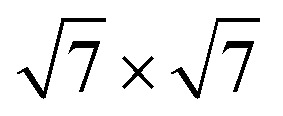
) overlayer structure at monolayer coverage, suggesting structural ordering at higher coverages.^122^ Importantly, the kinetic friction coefficient in the single-molecule limit was also quantified with *η* = 2.2 ± 0.2 ps^−1^, consistent with values obtained from corrected diffusion constants. Despite the weak vdW interaction between benzene and graphite, the friction is rather strong and is mainly attributed to phonon-mediated energy dissipation.^68^

More recent QENS measurements by Calvo-Almazan *et al.*^118^ for benzene adsorbed on graphite significantly extended the coverage range to 0.1–1.0 ML, suggesting that a revision of the simple diffusion model for this system is required. The study reveals that, in contrast to earlier assumptions of dominant surface friction, the primary source of kinetic friction arises from intermolecular interactions. At low coverage (0.1 ML), a super-diffusive regime was identified, characterised by Gaussian-shaped quasi-elastic scattering profiles indicative of ballistic motion (see also pyrene diffusion on graphite). As coverage increases, the shape of the scattering profiles transitions to Lorentzian, denoting a shift to Brownian diffusion. The transition correlates with a decrease in the mean free path and an increase in adsorbate-adsorbate interactions. The friction parameter for translational motion, *η*, as summarised in Table 1, increases markedly with coverage, ranging from 0.50 ps^−1^ at 0.1 ML to 1.82 ps^−1^ at 1.0 ML, while the diffusion coefficients correspondingly decreased.^118^ A rough hard disk (RHD) model, derived from a three-dimensional analogue, accurately describes the coverage-dependent friction up to 0.5 ML without fitting parameters. The model incorporates a cogwheel-like coupling of translational and rotational motion, accounting for angular momentum exchange during collisions. However, at full monolayer coverage (1.0 ML), the RHD model is no longer applicable, due to the prevalence of multi-body interactions and potential deviations from flat adsorption geometry upon transition to the multilayer regime.^122^ The total kinetic friction was successfully modelled as the sum of a constant surface friction component (≈0.3 ps^−1^) and a coverage-(*Θ*) and temperature-dependent collisional friction term, the latter scaling with 
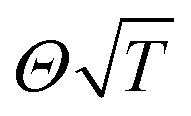
. These findings demonstrate the critical role of intermolecular forces in modulating surface diffusion under high coverage conditions with relevance for theoretical and simulation studies of molecular friction.^118^

### Summary

In summary, following Table 2, *E*_a_ for C_6_H_6_ diffusion clearly decreases when going from the Cu(100) surface with larger corrugation to the Cu(111) surface and the weakly interacting graphite. These findings illustrate how the symmetry and corrugation of the substrate fundamentally alter both the energetics and dynamics of molecular motion. Compared to Cu(100), the smoother PES of Cu(111) enables faster surface diffusion, with less pronounced contributions from rotational enhancement and frictional effects. Jump diffusion eventually turns into Brownian motion on graphite, while friction is strongly influenced by intermolecular interactions.^118,120,121^ As further shown, even long-range effects may be present,^123^ and give rise to repulsive signatures in diffusion.^74^

## Aromatic and heterocyclic ring systems

Beyond the prototypical case of benzene, aromatic and heterocyclic ring systems provide a structurally and electronically diverse set of adsorbates for studying molecule-surface interactions. Heterocyclic organic molecules, such as thiophene (C_4_H_4_S), pyrrole (C_4_H_5_N), pyrazine (C_4_H_4_N_2_), and s-triazine (C_3_H_3_N_3_), offer a particularly insightful platform due to the presence of heteroatoms like nitrogen and sulfur, which modulate both the electronic structure and adsorption geometry. These heteroatoms introduce local dipole moments and alter the polarisation of the π-system, resulting in adsorption behaviours distinct from those of homocyclic aromatics.

At the same time, the adsorption and diffusion of heterocyclic aromatics on metal surfaces are not only governed by binding energies but also by the intrinsic flexibility and anisotropy of the molecules. Experimental studies employing techniques such as HeSE and QENS have revealed unique dynamic behaviours – for example, enhanced rotational motion in C_4_H_5_N due to its polar N–H bond, and the directional bonding of C_4_H_4_S influenced by sulfur's electronegativity.

### Five-membered rings

Five-membered rings have so far mostly been studied on Cu surfaces, or more precisely on Cu(111). As summarised in Table 3, these include the 5-membered benzene analogue cyclopentadienyl (C_5_H_5_), as well as the heteroatomic molecules thiophene (C_4_H_4_S) and pyrrole (C_4_H_5_N).^66,124–126^ Early investigations using X-ray standing wave (XSW) and near-edge X-ray absorption fine structure (NEXAFS) spectroscopy showed that thiophene adsorbs flat on Cu(111) at low coverages, transitioning to a tilted geometry as coverage increases. The shift arises from a changing balance between π-bonding through the aromatic ring and σ-bonding *via* the sulphur lone pair.^127^ These studies have been pivotal for understanding the adsorption of larger thiophene derivatives with chiral, site-selective adsorption geometries governed by interactions between π-systems, lone-pair coordination, and the atomic registry of the top Cu layers.^128^ In a related context, pyrrole adsorption on Cu surfaces has been investigated due to its role in surface-selective inhibition. Shearer *et al.*^129^ investigated this interaction on clean and oxidised Cu(111) *via* experimental and DFT methods. On Cu(111), pyrrole adopts a planar geometry with an adsorption energy of −1.05 eV, forming a dense layer stabilised by dispersion forces. On CuO(111), it remains largely planar but slightly tilted due to surface corrugation, with a stronger adsorption energy of −1.10 eV, attributed to bonding with under-coordinated Cu atoms.^129^

**Table 3:** Summary of activation energies *E*_a_, friction coefficients *η*, and methods used for molecular diffusion studies of 5-membered rings on Cu(111) as shown in Fig. 10. All three molecules, cyclopentadienyl (C_5_H_5_), pyrrole (C_4_H_5_N) and thiophene (C_4_H_4_S) show activated jump diffusion, with some specific details for each system as further described in the text

Molecule	*E*_a_ (meV)	*η* (ps^−1^)	Methods	Ref.
C_4_H_4_S	20 (rot.)	5.0	HeSE, DFT & MD	66
	60 (transl.)			
C_4_H_5_N	50 ± 3	2.0	HeSE, DFT & MD	125
C_5_H_5_	40 ± 3	2.5	HeSE, DFT & MD	124

In terms of nanoscale dynamics, all three five-membered molecules exhibit activated jump diffusion characterised by comparable activation energies *E*_a_ for translational motion (Table 3). However, specific details of their diffusion dynamics diverge, as discussed below. Notably, rotational behaviour and quantum mechanical influences, such as the modulation of potential energy barriers by internal vibrational zero-point energies, strongly depend on the specific system.

#### Cyclopentadienyl

Cyclopentadienyl (C_5_H_5_), formed by the dissociative adsorption of cyclopentadiene (C_5_H_6_) on Cu(111) and studied by Hedgeland *et al.*,^124^ exhibits anionically adsorbed behaviour and is remarkably mobile despite its strong ionic binding to the surface. Using HeSE spectroscopy, the diffusion dynamics of 0.03 ML C_5_H_5_ was determined, revealing a jump diffusion mechanism between degenerate fcc and hcp hollow sites, evidenced by the two-component exponential decay in the ISF along different azimuths.^130^ An Arrhenius analysis of the dephasing rate yielded an effective activation energy of *E*_a_ = 41 ± 1 meV. The diffusion barrier obtained from DFT calculations closely matches the experimental results, confirming the bridge site as the transition state and the near-degeneracy of the fcc and hcp adsorption sites. A friction coefficient *η* = 2.5 ± 0.5 ps^−1^ was extracted by fitting Langevin MD simulations to the HeSE data, revealing strong coupling to substrate phonons. This frictional strength is comparable to that observed for benzene on graphite and significantly exceeds that found for alkali metals on Cu surfaces, underscoring the importance of phonon-mediated dissipation in organic adsorbates. Despite the ionic character and charge transfer, no long-range intermolecular interactions were observed, attributed to compensating effects such as the cushion effect and metal polarisability.^124^

#### Pyrrole

Lechner *et al.*^125^ combined HeSE spectroscopy and DFT to study pyrrole (C_4_H_5_N) diffusion on Cu(111), highlighting the influence of quantum effects in aromatics diffusion at low temperatures and sub-monolayer coverages. The experimental HeSE data revealed that pyrrole diffuses *via* activated jump diffusion, where the measured dephasing rates exhibit clear signatures of repulsive interactions between the molecules, even at low coverages. Diffusion occurs through jumps between bridge adsorption sites, with transition states located above fcc and hcp hollow sites. DFT calculations including dispersion corrections confirmed that the bridge site is the most energetically favourable adsorption site, but the computed classical barrier of ≈15 meV significantly underestimates the experimental activation energy of *E*_a_ = 50 meV.^125^

This discrepancy was resolved by considering the contributions of zero-point energy (ZPE) associated with internal molecular vibrations. Differences in ZPE between adsorption sites substantially increase the effective energy barrier, in particular, out-of-plane C–H and N–H bending modes and ring torsional modes, while high-frequency stretching modes contribute less. These ZPE differences raise the theoretical barrier to match experimental values, illustrating that the diffusion of pyrrole on Cu(111) is not governed solely by classical thermal activation, but significantly influenced by quantum mechanical effects through the modulation of potential energy barriers *via* internal vibrational zero-point energies.^125^

#### Thiophene

The surface diffusion dynamics of thiophene (C_4_H_4_S), a prototypical five-membered heterocycle, adsorbed on Cu(111) have also been investigated by Lechner and co-authors^66^ using HeSE measurements and DFT calculations. They identified three distinct thermally activated motions: jump diffusion between atop sites, rotation around the sulphur-copper anchor, and ring flapping between tilt states. As can be seen from the two slopes in the Arrhenius plot in Fig. 11, C_4_H_4_S is a typical example where rotational motion already sets in at lower temperatures due to the lower activation energy (Table 3) before translational motion starts to dominate at higher temperatures.

**Fig. 10:**
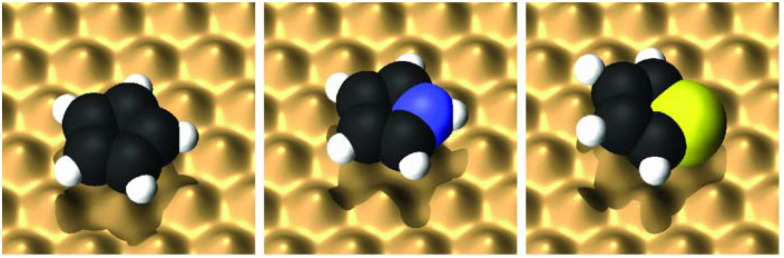
The three molecular five-membered rings, from left to right C_5_H_5_, C_4_H_5_N, and C_4_H_4_S on top of a potential energy surface. (Reprinted with permission from ref. 126, Copyright 2013 by AIP Publishing).

**Fig. 11:**
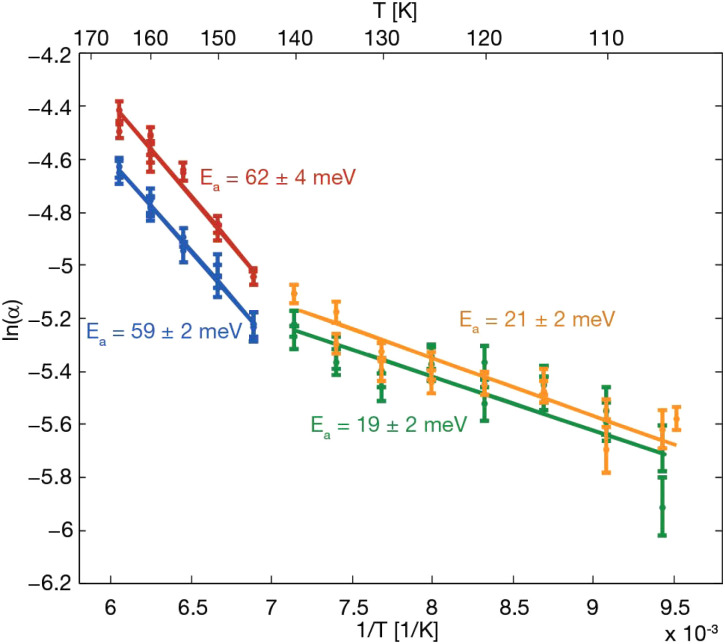
The Arrhenius plot from temperature-dependent measurements of C_4_H_4_S dynamics on Cu(111), taken at 0.7 Å^−1^ along the 
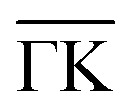
-azimuth, reveals two competing activated processes. The gradient gives an apparent activation barrier for rotation of 19 ± 2 meV at 0.015 ML (green) and 21 ± 2 meV at 0.022 ML (orange), while jump diffusion is more strongly activated with *E*_a_ = 59 ± 2 meV at 0.015 ML (blue) and *E*_a_ = 62 ± 4 meV at 0.022 ML (red). (Reprinted with permission from ref. 66, Copyright 2013 by the American Chemical Society).

Thiophene presents a higher diffusion barrier for translational motion (59–62 meV) and friction than similar five-membered aromatics such as cyclopentadienyl and pyrrole. Jump diffusion is dominant above 145 K and involves translations between adjacent atop sites *via* fcc and hcp transition states. Additionally, the system displays strong frictional coupling, with a high friction coefficient of 5 ± 2 ps^−1^, limiting the diffusive jump distance and ensuring predominantly single jumps.^66^ Rotations of the molecule about the sulphur anchor become dominant below 145 K, exhibiting an experimental barrier of 17 ± 2 meV and a DFT-estimated barrier of 29 meV. At intermediate temperatures and higher coverages, a third dynamic process, which involves ring flapping between adsorption geometries of differing tilt angles, was observed with a low activation barrier of 11 ± 2 meV. The study highlights the complex energy landscape experienced by thiophene on the surface, demonstrating that the adsorption and dynamics of heterocyclic aromatics on metal surfaces are not only governed by binding energies but also by the intrinsic flexibility and anisotropy of the molecule.

### Summary

In summary, comparing the diffusion of cyclopentadienyl (C_5_H_5_), thiophene (C_4_H_4_S) and pyrrole (C_4_H_5_N) (Fig. 10) as summarised in Table 3, provides a general picture of friction and diffusion in molecular adsorbates with different bonding configurations and adsorption geometries.^126^ Despite differences in adsorption sites - hollow for C_5_H_5_, bridge for C_4_H_5_N, and top site with tilting for C_4_H_4_ S – all three molecules exhibit high friction regimes, characterised by single jump diffusion between adjacent sites. The friction coefficients determined by HeSE experiments and corroborated by centre-of-mass and internal degrees-of-freedom MD simulations fall within the range of *η* = 2.0–5.0 ps^−1^, significantly exceeding those observed for atomic adsorbates. Notably, the friction is largely insensitive to adsorption strength or potential energy landscape corrugation, with internal degrees of freedom, in particular rotational modes around the surface normal, identified as the dominant contributors. For thiophene, which exhibits the largest friction, additional frictional contributions arise from frustrated rotational modes due to its tilted adsorption geometry because of the S-atom in the ring (Fig. 10). These findings underline the central role of molecular internal dynamics in dictating friction during surface diffusion for five-membered rings rather than molecule–substrate bonding strength, or site symmetry.^126^

### Heterocyclic six-membered rings

Nitrogen-containing heterocyclic molecules (see Table 1), including pyrazine (C_4_H_4_N_2_) and 1,3,5-triazine (s-triazine, C_3_H_3_N_3_), represent another important class of aromatic compounds. The adsorption and diffusion of heterocyclic molecules such as pyrazine and triazine are of growing interest for the functionalisation and doping of graphene and other graphitic materials, as well as for their potential in gas sensing applications.^131–133^ It is well established that the electronic properties of graphene can be tuned *via* non-covalent interactions with heterocyclic adsorbates. Reversible adsorption offers an effective strategy for chemical doping, and molecules such as triazine, pyrazine, and borazine have been shown to induce band gap opening in graphene.^132,134^ Significantly, the magnitude of this electronic modulation is strongly influenced by the electrophilic nature of the adsorbates.

Despite their technological relevance, their self-assembly behaviour and dynamics on carbon-based surfaces remain poorly characterised. Theoretical calculations suggest that, similar to benzene, these nitrogen-containing molecules adopt a flat, horizontally aligned adsorption geometry, with typical adsorption heights in the range of 3.00–3.21 Å.^135^ Experimental studies further confirm that pyrazine and triazine, lie flat on single-crystal metal surfaces,^136–138^ and STM investigations show that triazine also adsorbs parallel to HOPG.^137^ While the planar adsorption geometry is preserved across these molecules, the presence and number of nitrogen atoms significantly affect both the molecule–substrate interaction and the intermolecular interactions. Compared to benzene, the incorporation of nitrogen into the ring alters the distribution of electron density, thereby modulating vdW interactions and possibly introducing directional bonding effects.^132,135,139^

#### Pyridine

Pyridine (C_5_H_5_N) is somewhat unique among heterocyclic six-membered rings due to the presence of a single nitrogen atom, which breaks the rotational symmetry along the molecular *C* axis. This asymmetry leads to a preferred tilted adsorption geometry on surfaces, as described in several studies about its molecular orientation, bonding mechanisms, and dynamic behaviour on metal surfaces. Hou *et al.*^140^ used low-temperature STM and DFT to investigate pyridine adsorption on Ag(110). They identified two dominant configurations at low coverage: a parallel flat-lying geometry and a less frequent upright (perpendicular) mode. Both were governed primarily by electrostatic interactions between the nitrogen atom of pyridine and surface silver atoms, rather than strong covalent bonding. Tian *et al.*^141^ extended the investigation of pyridine derivatives by examining nicotinamide, pyridine-2-formamide, and pyridine-4-formamide on Pt(111) using DFT. All three molecules were found to chemisorb strongly in flat-lying orientations, forming C–Pt and N–Pt bonds with the surface. Adsorption energies varied based on the position of substituents, affecting the stability and electronic structure of the adsorbate-substrate system. Notably, the strongest binding was observed for nicotinamide.

García Rey *et al.*^143^ investigated the adsorption of pyridine (C_5_H_5_N) on Cu(110) using a combination of vibrational sum and difference frequency generation (SFG/DFG) spectroscopy, Kelvin probe work-function measurements, and DFT. This study aimed to understand how the molecular dipole of pyridine affects the local electric field at the metal interface and influences the nonlinear optical response. They found that a monolayer of pyridine reduced the Cu(110) work function by nearly 2.9 eV, one of the largest shifts reported for organic adsorbates. This was attributed to both charge transfer from the copper surface to the molecule and the alignment of molecular dipoles in the adsorbate layer. DFT calculations confirmed these findings, reproducing both the work-function shift and the enhanced dipole moment of adsorbed pyridine. SFG spectroscopy showed a substantial enhancement of the nonlinear optical signal with increasing pyridine coverage, whereas DFG was suppressed. The authors proposed that this contrasting behaviour results from dipole-moment reversal: when pyridine is excited by a 2.33 eV photon, charge moves from the nitrogen lone pair toward the ring, flipping the dipole direction. This dynamic dipole reversal modulates the local electric field and alters the nonlinear response. Pump–probe SFG experiments provided time-resolved confirmation of this effect. After excitation, the surface response dropped within ≈460 fs, indicating that the dipole-reversed excited state is stabilised by neighbouring molecules in the ground state, because it induces an electric field in the same direction as the ground state. The excited-state dipole persisted long enough to influence the interfacial electric field, demonstrating that photon-induced changes in molecular dipole orientation can be used to dynamically modulate the work function of metal–organic interfaces, with implications for tunable surface chemistry and optoelectronic device design.^143^

**Fig. 12:**
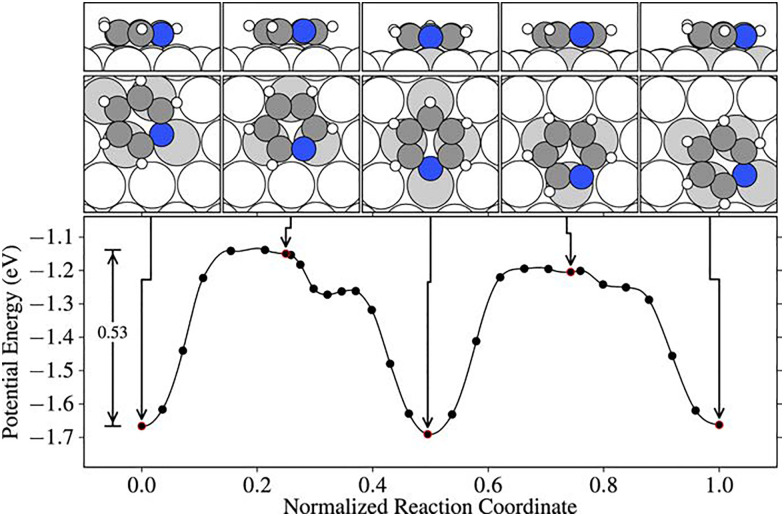
Energy profile for the lowest barrier diffusion path of pyridine on Pt(111) through the sequence bri-N_σ,fcc_ → fcc-N_σ_ → bri-N_σ,hcp_ → hcp-N_σ_ → bri-N_σ,fcc_. The path corresponds to stepwise rotations of the molecule around surface π-bonds, maintaining at least one strong C–Pt interaction throughout, and results in a diffusion barrier of 0.53 eV relative to the bri-N_σ,fcc_ minimum. (Reprinted with permission from ref. 142, Copyright 2016 by AIP Publishing).

Kolsbjerg *et al.*^142^ investigated the adsorption and diffusion of pyridine on Pt(111) using vdW-corrected DFT to understand its interaction with the metal surface at the atomic level. They found that pyridine adsorbs with its molecular plane nearly parallel to the surface, forming a tilted geometry where the nitrogen lone pair interacts directly with a Pt atom. The adsorption is stabilised primarily through a combination of covalent bonding at the nitrogen and dispersive interactions with the aromatic ring, yielding a calculated binding energy of −0.81 eV. The most favourable diffusion path involves the molecule moving laterally across the surface while maintaining its tilted geometry, as shown in Fig. 12. This motion is assisted by a pivot-like interaction of the ring over a single Pt atom, resulting in a diffusion barrier of 0.53 eV. The preferred pathway can be linked to the relative strength of the C_2_–Pt π-bond compared to other possible bonding motifs involving the nitrogen atom, influencing adsorption orientation and mobility.

These studies demonstrate that pyridine and its derivatives interact with metal surfaces through a balance of weak covalent, dispersive, and electrostatic forces. Molecular orientation – whether flat or tilted -emerges as a key determinant of surface mobility and diffusion barriers.

#### Pyrazine

**Fig. 13:**
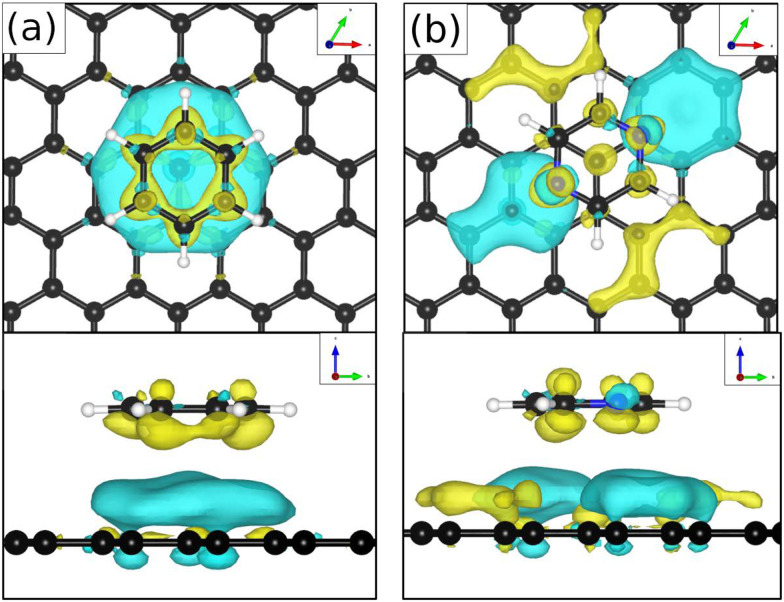
(a) and (b) show a top and side view of the energetically most favourable adsorption geometry of C_6_H_6_ and C_4_H_4_N_2_ on graphite(0001), respectively, together with the charge density distribution based on vdW corrected DFT calculations. Charge accumulation and charge depletion with respect to isolated benzene and pyrazine, are shown in yellow and blue, respectively, with the isosurface cutoff being set to 0.01 e Å^−3^ (Reprinted from ref. 99 under the terms of the Creative Commons CC BY license).

Pyrazine and triazine have been studied for their adsorption and diffusion on surfaces due to their structural similarity to benzene, but with nitrogen atoms incorporated into the aromatic ring. These heteroatoms introduce additional electronic functionality, such as lone-pair interactions and altered dipole moments, that influence adsorption strength, orientation, and surface interactions. Compared to benzene, which interacts primarily through delocalised π–π and vdW forces, pyrazine and triazine can engage in more complex binding *via* nitrogen–metal coordination or electrostatic interactions (see Fig. 13). Studies using STM, neutron scattering, and DFT have shown that these heterocycles often form more stable and ordered surface phases and exhibit lower mobility, due to stronger molecule–substrate interactions and directional bonding.^99,136,144–148^ It makes them useful models for tuning molecular self-assembly and surface functionalisation in catalysis, molecular electronics, and selective adsorption applications.

Wang *et al.* used STM measurements to investigate the adsorption and self-assembly of three nitrogen-containing aromatic molecules – pyridine, pyrazine, and triazine – on Cu(111) surfaces under electrochemical conditions.^136^ All three molecules were found to adsorb in the double-layer potential region and form well-ordered adlayers with a (3 × 3) periodicity, corresponding to a uniform surface coverage of 0.11 ML. The molecules adopt a flat-lying orientation on the surface, stabilised by π-electron interactions with the substrate. While the STM images clearly showed long-range structural ordering for each compound, the study did not directly measure or compare molecular mobility or diffusion. The results suggest that increasing the number of nitrogen atoms may enhance molecule–substrate interactions through additional lone pair coordination, which could contribute to the stability of the observed adlayers. However, no explicit differences in structural stability or molecular dynamics were quantified.

On the other hand, Maier *et al.*^99^ investigated how vdW interactions influence the adsorption structure and stability of aromatic molecules on graphite surfaces. Using neutron diffraction and vdW-corrected DFT, they compared the adsorption of deuterated pyrazine (C_4_H_4_N_2_) to that of benzene (C_6_H_6_) on the graphite (0001) basal plane, as shown in Fig. 13. The study revealed that pyrazine forms a more thermodynamically stable overlayer, maintaining structural integrity up to 320 K and exhibiting continued layer-by-layer growth. While Maier *et al.* did not provide explicit diffusion parameters for pyrazine on graphite, additional NSE measurements indicate reduced mobility and higher thermal stability due to stronger molecule–substrate and intermolecular interactions at higher coverages.^99,149^ This behaviour is attributed to the nitrogen atoms in pyrazine, which enhance polarisability and strengthen van der Waals interactions with the graphite substrate, as well as stronger inter-molecular interaction compared to benzene.

#### Triazine

s-Triazine (C_3_H_3_N_3_) has been studied on graphite^137^ and graphene/metal surfaces, namely on graphene/Pt(111)^150^ and graphene/Rh(111),^151^ as summarised in Table 4. Dynamics data was extracted from STM measurements based on island densities at different temperatures, and even though all three interfaces exhibit the same structure there are clear differences.

**Table 4:** Activation energies *E*_a_ for s-triazine (C_3_H_3_N_3_) on various substrates, as determined from island size distributions in STM measurements, illustrate the effect of the supporting metal substrate underneath graphene on *E*_a_

Substrate	*E*_a_ (meV)	Methods	Ref.
Graphite	55	STM & DFT	11,137
Graphene/Pt(111)	68	STM & DFT	11,150
Graphene/Rh(111)	80	STM	151

The initial stages of s-triazine (C_3_H_3_N_3_) adsorption on HOPG at temperatures below 100 K were investigated using variable-temperature STM, revealing the formation of monolayer, dendritic islands composed of flat-lying molecules arranged in a hexagonal lattice with a periodicity of 6.1 Å.^137^ Analysis of nucleation behaviour determined a critical nucleus size of one, indicating that dimers are stable against dissociation. An identical critical nucleus size was found for other graphene/metal systems^150,151^ as further described below, despite clear differences in terms of the dynamics. From the temperature-dependent island densities and application of nucleation theory, a diffusion barrier of 55 ± 8 meV and an attempt frequency of 1 ⋅ 10^14^ s^−1^ were extracted for single-molecule diffusion. While the barrier suggests a weak molecule–substrate interaction typical of physisorption, it is significantly higher than for benzene on HOPG, implying that nitrogen substitution in the triazine ring enhances bonding with the substrate, likely due to altered π-orbital interactions.^137^

In a similar manner, the diffusion behaviour of s-triazine on graphene supported by metal substrates has been investigated through STM and nucleation studies on both weakly and strongly interacting systems. On graphene/Pt(111), triazine molecules form well-ordered hexagonal islands with a lattice constant of 6.25 Å, lying flat on the surface due to physisorption *via* π-orbital interactions and the diffusion barrier was measured as *E*_a_ = 68 ± 9 meV. This is higher than the 55 meV found on graphite, evidencing the influence of the metallic substrate, albeit being a weakly interacting one.^150^

Finally, on graphene/Rh(111), a system with stronger graphene-metal interaction, triazine also adsorbs flat, forming a hexagonal lattice with a periodicity of 6.3 Å. Despite the stronger substrate interaction, the molecule–substrate bonding remains physisorptive, as confirmed by the diffusion barrier *E*_a_ = 80 ± 9 meV. Notably, different molecular orientations with respect to the graphene lattice are observed, attributed to varying Moiré domains. Diffusion was found to be influenced by the Moiré pattern, which gives rise to higher activation energies and thus lower diffusivity, which becomes more pronounced, likely with increasing graphene-metal coupling.^151^

### Summary

In summary, as shown in Table 4, s-triazine diffusion on metal-supported graphene is characterised by increased energy barriers relative to graphite, reflecting the influence of substrate-induced modulation *via* Moiré patterns and graphene-metal coupling. While diffusion on weakly interacting graphene/Pt(111) proceeds with low activation energy, stronger coupling on graphene/Rh(111) introduces local variations in the adsorption landscape. Nevertheless, across all systems, triazine exhibits physisorptive, flat-lying adsorption and a consistent critical nucleus size (*i* = 1), suggesting that intermolecular interactions dominate nucleation behaviour. These results, in line with broader findings from ref. 11, highlight the potential of graphene-metal substrates as tunable templates for diffusion-controlled molecular architectures. The observed trend that substituting carbon with nitrogen in the aromatic ring, as also evidenced by the increased thermal stability of pyrene films,^99^ enhances the film stability of nitrogen-containing aromatics on graphene and graphite, merits further exploration. However, the relative contributions of intermolecular *versus* molecule–substrate interactions remain ambiguous, given that existing studies rely on indirect measures such as island size distributions. Direct dynamical measurements are thus essential, and QENS data of pyrazine and s-triazine diffusion on exfoliated graphite may offer critical insight into these fundamental processes.^149,152^

## Polycyclic aromatic hydrocarbons

Polyaromatic hydrocarbons (PAHs) such as pentacene are prototypical π-conjugated systems widely used in organic semiconductors and thin film growth, owing to their well-characterised structural and electronic properties.^56–58,153–155^ Despite extensive studies of post-growth morphology and film structure,^117,154,156–160^ direct investigations of the atomic-scale dynamics of PAHs during adsorption, diffusion, and nucleation remain comparatively scarce.^10^ Such dynamics, however, govern the kinetic pathways of self-assembly and influence the quality of the resulting thin films.

In the sub-monolayer regime, the adsorption geometries of many PAHs closely resemble that of benzene, typically adopting flat-lying configurations to maximise π-metal or π-surface interactions (see Fig. 7(b) for pyrene on graphite).^67,161^ For example, naphthalene preferentially adsorbs in a planar orientation on Pt(111), with an adsorption energy of −2.81 eV and a mean Pt–C separation ranging from 2.08 to 2.25 Å.^162^ As illustrated by Björk *et al.*, using dispersion-corrected DFT methods, the adsorption of PAHs on graphite occurs in an AB-stacking configuration analogous to that in graphite (see Fig. 7(b)). The adsorption energy per atom exhibits a linear dependence on the hydrogen-to-carbon ratio, N_H_/N_C_, which permits a decomposition into graphene-like (*E*_CC_) and benzene-like (*E*_CH_) carbon contributions. The TS correction yields *E*_CC_ = 74.9 meV and *E*_CH_ = 95.8 meV per C-atom in the molecule, revealing stronger binding at benzene-like sites due to their distinct local chemical environment.^161^ While this size-dependent enhancement implies stronger adsorption for larger PAHs, as confirmed by experimental observations,^103,117^ it does not necessarily give rise to a corresponding decrease in molecular mobility as demonstrated for selected systems below.

### Naphthalene

Kolsbjerg *et al.*^162^ explored the adsorption and molecular dynamics of naphthalene (C_10_H_8_) molecules on a Pt(111) surface using a combination of STM and vdW corrected DFT calculations. Diffusion across the Pt(111) surface involves activated jump diffusion between multiple local minima in a complex energy landscape, where naphthalene undergoes translational diffusion through a network of small-step transitions. The lowest energy diffusion pathway has a computed activation barrier of 0.78 eV, with alternative competitive paths within a 0.1 eV window. All effective pathways involve intermediate local minima, particularly the second most stable configuration.^162^

STM imaging between 263 K and 301 K showed frequent on-site rotational events, interpreted as ±60° or ±120° rotations due to naphthalene's symmetry. The experimentally determined activation energy for rotation is 0.67 ± 0.07 eV, while DFT calculations predict a rotation barrier of 0.75 eV. The theoretical pre-exponential factor calculated from harmonic transition state theory (HTST) is 1.04 ⋅ 10^13^ s^−1^, whereas the experimental prefactor derived from Arrhenius fitting is 4 ⋅ 10^9^ s^−1^, highlighting the limitations of HTST for rotational dynamics. Translational diffusion events are less frequent than rotations, suggesting a lower probability due to higher activation energy or reduced vibrational accessibility. Theoretical analysis within HTST and Langevin dynamics frameworks suggests significant frictional damping and anharmonic contributions not captured in standard DFT-based prefactor estimates.^162^

### Pyrene

A very interesting example for PAHs is pyrene (C_16_H_10_), whose dynamics on graphite were investigated through a combination of QENS, MD and DFT simulations.^67^ At sub-monolayer coverages (0.1–0.2 ML), pyrene undergoes ballistic diffusion – a regime where molecules move quasi-freely over the surface with negligible energy barriers.^67^ This regime is rare and, prior to this study, had only been observed for a physisorbed gas (Xe on Pt(111)) at cryogenic temperatures.^163^

**Fig. 14:**
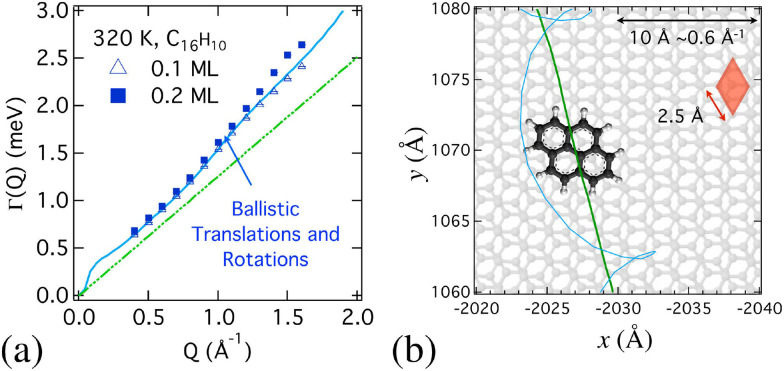
Pyrene (C_16_H_10_) diffusion on graphite follows ballistic diffusion. (a) Quasi-elastic broadening *Γ* as a function of momentum transfer *Q* from neutron TOF measurements for 0.1 ML (triangles) and 0.2 ML (squares) pyrene on graphite. The linear dependence is characteristic of ballistic diffusion, while the finite intercept indicates additional contributions from molecular rotation. (b) Schematic representation of the molecular dynamics from simulations: the green line shows the ballistic centre-of-mass motion of the pyrene molecule, while the blue trajectory illustrates the rotational motion of a single hydrogen atom. (Reprinted with permission from ref. 67, Copyright 2016 by the American Chemical Society).

The scattering functions derived from neutron TOF data exhibit Gaussian lineshapes, a characteristic feature of ballistic motion,^118^ in contrast to Brownian motion, which yields Lorentzian lineshapes and a parabolic dependence of *α* on Δ*K* as is the case for the benzene/graphite scenario. The extracted quasi-elastic broadening *Γ* as a function of momentum transfer Δ*K* or *Q* – which is the more common notation in QENS – is shown in Fig. 14(a). The quasi-elastic broadening *Γ* clearly follows the linear behaviour as a function of Δ*K*, which is characteristic for the ballistic regime according to:7
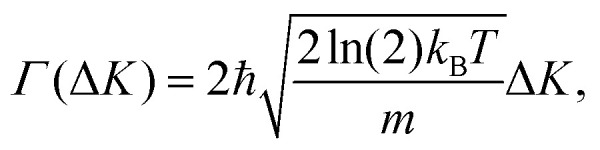
with the surface temperature *T* and the adsorbate mass *m*. Hence, the slope of the linear behaviour in terms of Δ*K* is directly proportional to *T* and inversely proportional to the adsorbate mass *m*. The C_16_H_10_ data measured at 320 K clearly follows the expected linear slope in Fig. 14(a) while a finite offset reflects additional rotational motion of the molecule. This is reflected in the real space motion according to MD simulations as shown in Fig. 14(b), where the centre of mass (CoM) of C_16_H_10_ follows the expected ballistic motion along a straight line and the H-atoms of C_16_H_10_ simultaneously undergo rotations.

DFT calculations further reveal very low diffusion barriers (≈11 meV), and the mentioned MD simulations help to separate translational and rotational components of the motion. The mean free path and observed dephasing rates support a model in which molecular collisions and internal rotational degrees of freedom dominate the frictional dissipation, rather than substrate interactions. These findings suggest that in dilute PAH systems on graphite, kinetic friction can be significantly reduced or even vanish, enabling nanoscale superlubricity. Comparative studies with benzene under similar conditions show a transition to Brownian diffusion, highlighting the influence of molecular size and coverage on diffusion behaviour.^67^

### Pentacene

As mentioned above, pentacene in particular has been extensively studied, due its potential use for organic semiconductors. However, these studies concentrate on the post-growth morphology and film structure.^154–157,164–166^ In contrast, the only direct measurement of pentacene (C_22_H_14_) surface diffusion has been reported by Rotter *et al.*, for C_22_H_14_ diffusion on an organic pentacene monolayer adsorbed on Cu(110).^10^

**Fig. 15:**
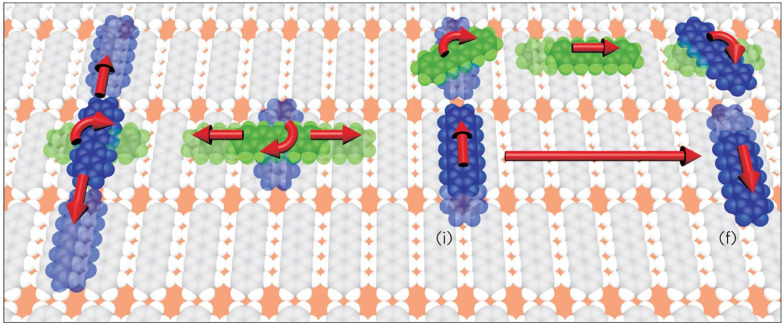
Illustration of C_22_H_14_ diffusion on C_22_H_14_/Cu(110) illustrating single admolecules in potential energy minimum positions and the corresponding elementary diffusion processes. C_22_H_14_ molecules preferentially move along the direction of their long axis (straight arrows, blue along 
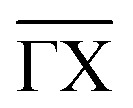
, green along 
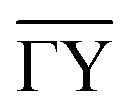
), but sometimes turn 90° (curved arrows). The right-hand side shows a molecule that initially (position (i)) diffuses along 
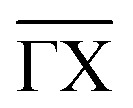
 until it turns 90°, moves along 
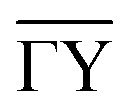
 and finally (position (f)) turns back. The latter appears as a long jump along the 
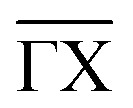
 azimuth (long red arrow), covering multiple lattice distances, while it involves six individual steps. (Reprinted with permission from ref. 10 Copyright 2016 by Springer Nature).

HeSE measurements around room temperature reveal a complex motion dictated by a coupling between molecular translation and rotation as illustrated in Fig. 15: adparticles preferentially occupy symmetry-specific adsorption sites of the underlying monolayer and diffuse along orthogonal “rails” aligned with the molecular C_22_H_14_ axes or the 
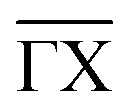
-azimuth of the underlying Cu(110) substrate. Diffusion proceeds through long jumps facilitated by rotational events that allow transitions between these rails. The effective activation energies for diffusion were determined to be 93 ± 9 meV along 
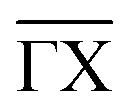
 and 101 ± 1 meV along 
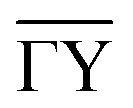
, correlating with rotation-mediated rerouting between energy minima.

Langevin simulations provided further insight into the energy landscape, revealing rotation barriers of around 110 meV and inter-rail barriers exceeding 185 meV, consistent with the experimentally observed long-jump behaviour. Tracer diffusion coefficients were extracted as 3.3 ⋅ 10^−9^ m^2^ s^−1^ and 2 ⋅ 10^−9^ m s^−1^ for 
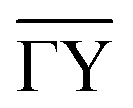
 and 
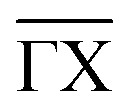
, respectively. A friction coefficient *η* = 1.8 ± 0.2 ps^−1^ was derived, affirming the overdamped regime of pentacene motion on the organic substrate.^10^

### Summary

In summary, studies on the dynamics of polycyclic aromatic hydrocarbons (PAHs) have unveiled a diverse range of motions, from the ballistic transport of pyrene to the anisotropic jump-diffusion of pentacene. Together, these findings demonstrate the role of PAHs as ideal model systems for probing friction, diffusion, and energy dissipation in weakly interacting interfaces-critical for advancing nanotribology and molecular-scale device design. However, despite theoretical insights into how adsorption characteristics evolve with PAH size, direct experimental investigations remain scarce, with much of the existing work focusing instead on long-range structural ordering^167^ or intermolecular interactions such as stacking.^168,169^

## Other and more complex organic molecules

Although not the primary focus of this review, numerous foundational insights into surface diffusion have been derived from studies on large organic molecules adsorbed on metal substrates. Owing to their greater mass and typically stronger molecule–substrate interactions, such systems exhibit reduced mobility, enabling direct observation of diffusion events *via* STM at low temperatures. For completeness, we briefly summarise these findings to contextualise the broader landscape of molecular diffusion phenomena and to highlight methodological developments that have proven instrumental in the study of more weakly interacting systems such as aromatics on graphene and graphite.

In addition to pentacene, which is often regarded as a prototypical system for organic thin-film growth, rod-like molecules such as para-hexaphenyl (6P, C_36_H_26_) have also been investigated for their self-assembly and thin-film growth mechanisms. While 6P has received comparatively less attention compared to pentacene, it remains a relevant material in organic electronics, owing to its anisotropic structure and favourable semiconducting properties. Studies have shown that substrate-dependent molecular orientation and packing significantly affect the resulting film morphology and functional performance.^154,170–172^ Nevertheless, current understanding is predominantly based on post-growth analyses such as island size distributions and growth kinetics, with a notable absence of direct measurements capturing real-time diffusion dynamics.

Moving beyond simple PAHs, more complex π-conjugated molecules have been investigated, revealing a wide spectrum of diffusive behaviours and the involved potential energy surfaces, as summarised in Table 5. These studies, predominantly conducted *via* STM, highlight the sensitivity of surface mobility to subtle changes in molecular architecture. Note here that diffusion, as introduced in eqn (2), remains observable even for activation energies near 1 eV, as reported in Table 5, due to the large attempt frequencies (*ϒ*_0_ in eqn (1) ≈ 10^12^–10^14^ s^−1^) entering the Arrhenius prefactor. Despite such barriers being much higher than the thermal energy at room temperature (*k*_B_*T* ≈ 25 meV), the Boltzmann-weighted probability of accessing the transition state, expressed by the exponential factor in *ϒ* = *ϒ*_0_exp(−*E*_b_/*k*_B_*T*), ensures non-negligible hopping rates. Additionally, collective effects in dense films and tip-induced perturbations in STM experiments can further enhance apparent mobility. We start by examining a set of “linear” polyaromatics whose core structures are modified by the presence of functional groups, such as thiols, which affect adsorption configurations and dynamic response on surfaces.

**Table 5:** Summary of experimentally obtained activation energies *E*_a_ for the surface diffusion of larger and more complex π-conjugated molecules

Surface	Molecule	Mass(amu)	*E*_a_ (eV)	Exp.	Ref.
Pd(110)	C_12_H_9_NO_2_	199.1	0.83	STM	77
Cu(111)	C_14_H_10_S_2_	242.0	0.13	STM	78
Cu(111)	C_14_H_8_O_2_	208.1	0.02	STM	17
Ag(100)	C_32_H_16_CoN_8_	571.1	0.15 (low-T)	STM	18
			0.1 (high-T)	HeSE	93
Cu(111)	C_44_H_30_N_4_	614.2	0.71 (diff)	STM	82
			1.28 (rot)		
Cu(111)	C_40_H_26_N_8_	618.2	0.96	STM	81
Cu(110)	C_36_H_18_	450.1	0.74	STM	80
Cu(110)	C_60_H_66_	786.5	0.57	STM	80
Graphite	C_18_H_15_P	262.1	0.05	QENS	88
Cu(111)	C_33_H_24_IrN_3_	654.4	0.20	STM	173
Pd(110)	C_60_	720.0	1.40	STM	79
CaF_2_(111)	C_60_	720.0	0.21	AFM	83

For example, the diffusion of 4-[*trans*-2-(pyrid-4-yl-vinyl)] benzoic acid (C_14_H_11_NO_2_, PVBA) on Pd(110) proceeds strictly one-dimensionally along the close-packed [001] direction, as directly observed *via* STM measurements. The molecules adsorb flat, bridging three Pd atomic rows diagonally, leading to four equivalent adsorption configurations. Surface diffusion follows an Arrhenius behaviour with an activation energy of *E*_a_ = (0.83 ± 0.03) eV and an attempt frequency of 10^10.3±0.4^ s^−1^. Assuming single nearest-neighbour hopping, the one-dimensional diffusion coefficient prefactor is *D*_0_ = 7.55 ⋅ 10^−10^ m^2^ s^−1^.^77^

Low-temperature STM and DFT studies show that 9,10-dithioanthracene (C_14_H_8_S_2_) exhibits unidirectional diffusion along the high-symmetry axes of the Cu(111) surface, with an activation energy of 130 meV. The molecular design, featuring two thiol linkers, constrains rotation and enforces linear motion *via* a “walking” mechanism, where alternating substrate anchoring prevents lateral deviation or rotation, which highlights the role of molecular geometry and bonding configuration in directional surface transport.^78^

The diffusion of anthraquinone (C_14_H_8_O_2_, AQ) on Cu(111) is strictly linear along high-symmetry directions, even at temperatures as low as 20 K and exhibits an activation energy as low as ≈23 meV. Upon attachment of one and two CO_2_ molecules, the diffusion barrier increases incrementally by approximately 0.03 eV and 0.02 eV, respectively. Despite the attached CO_2_ molecules, AQ retains its characteristic linear motion, effectively acting as a molecular carrier.^17^

**Fig. 16:**
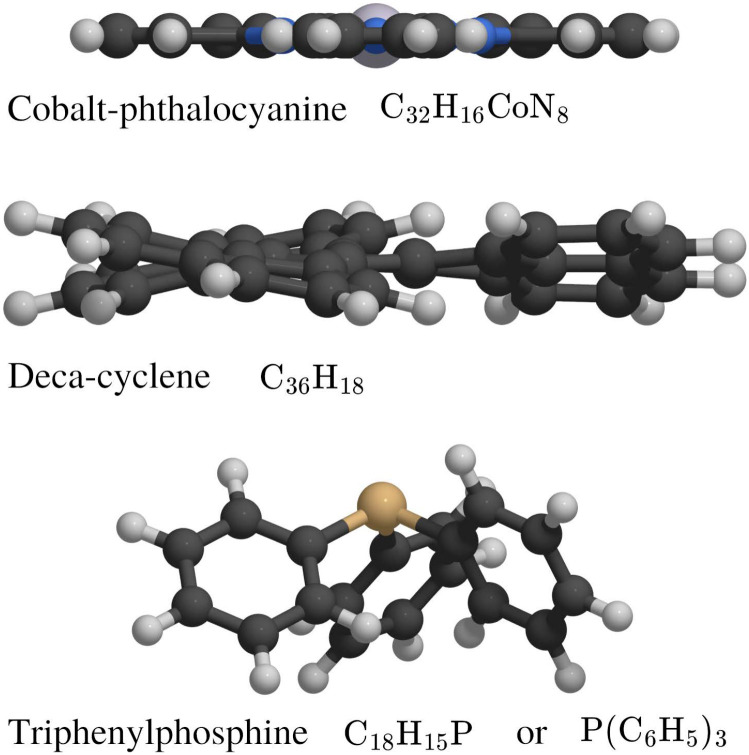
The side view of several π-conjugated molecules illustrates how their geometry changes from planar (C_32_H_16_CoN_8_) to more complex geometries such as the twisted C_36_H_18_ and the pyramidal C_18_H_15_P.

Progressing towards larger and “heavier” molecules listed in Table 5, we encounter systems ranging from highly planar structures, such as phthalocyanines, to more complex architectures that deviate from planarity (see Fig. 16). In these latter cases, the interplay between molecular conformation and surface interaction becomes increasingly significant. Such non-planar geometries can lead to conformational flexibility, introducing internal degrees of freedom that may couple with translational motion. This coupling can manifest as conformational rearrangements concurrent with diffusion, complicating the mechanistic picture and demanding advanced experimental and theoretical approaches to disentangle these dynamic processes.^5,174^

*E.g.*, the interesting properties of planar larger molecules such as phthalocyanine (Pc's) have triggered a number of studies focusing on their use in electronics, sensors,^175–177^ quantum computing,^178,179^ and magnetic moment studies.^180^ Investigations span from quasi-isolated molecules at cryogenic temperatures^181^ to molecules immobilised within molecular layers at ambient or elevated temperatures.^18,182^ The surface diffusion of Cobalt phthalocyanine (CoPc, C_32_H_16_CoN_8_) on Ag(100) was investigated at cryogenic temperatures (43–50 K) *via* STM. The molecule exhibits both translational and rotational motion, with translation dominating by a factor of four. The measured activation energy for diffusion is *E*_a_ = 0.15 ± 0.01 eV and the prefactor *D*_0_ ≈ 1 ⋅ 10^−5^ m^2^ s^−1^, consistent with a hopping mechanism between hollow sites and infrequent rotations, indicating coupled motion dominated by translation.^18^ DFT calculations confirm preferential adsorption at hollow sites with an adsorption energy of 6.21 eV and a planar geometry. At elevated temperatures (250–350 K), Sabik *et al.* used HeSE to probe CoPc diffusion on picosecond timescales. They observed a reduction in the activation barrier to ≈100 meV and a transition to predominantly long jumps spanning multiple lattice sites. The Chudley-Elliott model captures this behaviour, with a molecular residence time of 0.11 ns at 350 K and a diffusion coefficient *D* ≈ 9.6 ⋅ 10^−10^ m^2^ s^−1^. The derived diffusivity prefactor is *D*_0_ ≈ 2.7 ⋅ 10^−8^ m^2^ s^−1^ and thus significantly smaller than in the low-temperature STM study.^93^ Additionally, they characterised CoPc diffusion as a low-friction process, influenced by internal molecular degrees of freedom. While molecular flexibility contributes to the observed dynamics, these findings highlight that surface diffusion mechanisms evolve significantly across the temperature range, and the assumption of uniform behaviour from cryogenic to elevated temperatures is not generally valid.^93^

2H-tetraphenylporphyrin (C_44_H_30_N_4_, 2HTPP), on the other hand, can no longer be considered as perfectly planar. The diffusion of 2HTPP on Cu(111), measured between 280 and 345 K, exhibits predominantly unidirectional diffusion along the close-packed [1̄10] directions of the substrate. The activation energy for this translational motion is *E*_a_ = 0.71 ± 0.08 eV, while a higher barrier of *E*_a_ = 1.28 ± 0.12 eV was determined for the reorientation of the diffusion direction. Here, strong localisation and reduced mobility are attributed to specific coordinative bonding between iminic nitrogen atoms and the copper surface.^82^

Similarly, the diffusion behaviour of tetrapyridylporphyrin (C_40_H_26_N_8_, TPyP) on Cu(111) studied by Eichberger *et al.* in the 300–360 K range,^81^ shows that individual TPyP molecules undergo strictly unidirectional one-dimensional (1D) thermally activated diffusion along the [1̄10] direction, guided by a saddle-shaped conformational adaptation to the surface. The monomer diffusion exhibits an activation energy of *E*_a,m_ = 0.96 ± 0.09 eV with a prefactor *ϒ*_0_ = 1.4 ⋅ 10^12^ s^−1^. Interestingly, equally oriented molecules form dimers with drastically enhanced 1D mobility, despite a similar activation energy (*E*_a_ = 0.94 ± 0.03 eV). The increase is attributed to a higher prefactor (*ϒ*_0,d_ = 1.9 ⋅ 10^14^ s^−1^), indicative of an entropically favoured collective motion mechanism. These dimers are proposed to form *via* coordination with thermal Cu adatoms, yielding a metallosupramolecular complex that facilitates rapid diffusion.^81^

By contrast, decacyclene (DC, C_36_H_18_), as also shown in Table 1 and Fig. 16, exhibits a clearly twisted molecular geometry, while hexa-*tert*-butyl decacyclene (HtBDC, C_60_H_66_) can be considered to exhibit almost a propeller-like structure. Following STM measurements, the diffusion of DC and HtBDC on Cu(110) are dominated by long jumps, with jump lengths of 3.9 ± 0.2 and 6.8 ± 0.3 lattice spacings, respectively. Both molecules adsorb *via* their aromatic π-systems and diffuse one-dimensionally along the [001] direction. The activation energies for diffusion are 0.74 ± 0.03 eV for C_36_H_18_ and 0.57 ± 0.02 eV for C_60_H_66_. The corresponding prefactors are *ϒ*_0_ = 10^13.9±0.7^ s^−1^ and *ϒ*_0_ = 10^13.5±0.4^ s^−1^, while the diffusion coefficient increases almost by a factor of 10^4^ for C_60_H_66_. This enhancement is attributed to its reduced substrate coupling due to the bulky *tert*-butyl groups, which also reduce frictional resistance to motion.^80^

A similar structure can be ascribed to the propeller-like tris(2-phenylpyridine)iridium, (C_33_H_24_IrN_3_, Ir(ppy)_3_) molecules. The surface diffusion of Ir(ppy)_3_ on Cu(111) exhibits an activation energy of *E*_a_ = 203 ± 7 meV, and a pre-exponential factor *ϒ*_0_ = 10^11.3±0.6^ s^−1^. These kinetic parameters indicate relatively weak molecule–substrate interactions, attributed to the non-planar, propeller-shaped conformation of Ir(ppy)_3_. The calculated diffusion coefficient is *D*_0_ = 3.6 ⋅ 10^−9^ m^2^ s^−1^, lower than typical metal adatom systems due to the complexity and flexibility of the diffusing species.^173^

It is interesting to compare the aforementioned systems with one of the very few studies conducted in reciprocal space within this category, namely that of triphenylphosphine (C_18_H_15_P). Despite being smaller in overall size compared to some of the previously discussed molecules, triphenylphosphine adopts a pyramidal geometry (P(C_6_H_5_)_3_), as shown in Fig. 16, and has been described as a nanoscopic “moonlander”.^88^ As seen in QENS studies on exfoliated graphite, translational mass transport is significantly influenced by the internal dynamics of the phenyl rings. These rotational modes become active even at low temperatures and contribute to a complex dynamical profile. Remarkably, although the molecule exhibits a comparatively high adsorption energy, the translational diffusion barrier remains modest at just 46 meV, suggesting that the internal degrees of freedom and in particular the mobility of the phenyl groups, can effectively facilitate surface diffusion.^88^

Finally, the comparison of diffusion parameters for larger π-conjugated molecules, as summarised in Table 5, can be extended by considering the case of the fullerene C_60_, a cage-like molecule with a highly delocalised π-system. The diffusion of C_60_ on Pd(110) exhibits thermally activated behaviour with a high tracer diffusion barrier of 1.4 ± 0.2 eV and an unusually large pre-exponential factor of 10^14.4±0.4^ s^−1^, determined from STM measurements in the temperature range 435–485 K. The motion is interpreted as a rolling mechanism, which retains high C–Pd coordination and may account for the large prefactor and reduced friction. Upon annealing to ≈700 K, C_60_ undergoes a bonding transition, becoming embedded in Pd surface pits with increased coordination and substantially reduced mobility.^79^

The diffusion of C_60_ was further analysed on the insulating CaF_2_(111) surface under ultra-high vacuum using the onset method, based on island nucleation statistics derived from non-contact AFM measurements. Due to the weak molecule–substrate interaction, direct observation of individual hopping events was not feasible. From the temperature-dependent island densities, a diffusion barrier of *E*_a_ = 214 ± 16 meV and an attempt frequency of *ϒ*_0_ = 1.4 ⋅ 10^12±0.6^ s^−1^ were extracted. The deduced parameters are consistent with the expected low friction and high mobility for C_60_ on wide bandgap insulators, and much lower than values reported for metal surfaces.^83^

In addition to systematic quantitative studies, more exploratory approaches have also been pursued, including molecular machines such as nanocars and walkers,^183–185^ as well as purely theoretical investigations based on DFT.^186^ Among the earliest STM studies on large π-conjugated systems, Violet Lander (C_108_H_104_) molecules on Cu(110) exemplify how diffusion can be dramatically influenced by molecular orientation. In a “lock-and-key” configuration aligned with the substrate, the molecules are effectively immobilised, whereas a rotated orientation yields a diffusion coefficient two orders of magnitude higher, from *D* < 5 ⋅ 10^−23^ m^2^ s^−1^ to *D* = (4.8 ± 0.5) ⋅ 10^−21^ m^2^ s^−1^ at 180 K.^16^

As we have seen from the examples above, in the context of larger π-conjugated molecules, surface diffusion behaviour becomes increasingly complex due to the possibility of multiple adsorption conformations. These conformational states are not solely thermally accessible but can also be deliberately induced through external perturbations, including tip-molecule interactions in scanning probe experiments. Tip-induced conformational switching offers a means to dynamically manipulate molecular states, providing insight into energy barriers and transition pathways. Moreover, long-range diffusion events facilitated by such interactions illustrate the potential to steer molecular motion beyond thermal activation.

For instance, Cao *et al.* investigated the behaviour of diphenylcarbene (C_13_H_10_, DPC) on Cu(111), focusing on chirality control *via* tip-induced vdW interactions.^187^ DPC exhibits two enantiomeric forms due to its twisted phenyl rings, which can be reversibly interconverted using inelastically tunnelling electrons. Strong covalent anchoring of the carbene centre to the substrate suppresses translational diffusion, resulting in a system where only intramolecular rotation governs dynamic behaviour.^187^ In contrast, Civita *et al.* demonstrated long-range surface diffusion of dibromoterfluorene (C_21_H_14_Br_2_, DBTF) on Ag(111) at cryogenic temperatures below 7 K.^188^ Upon alignment along the 〈11̄0〉 direction, molecules accessed a high-mobility regime characterised by one-dimensional motion confined to atomic rows. This anisotropic diffusion, electrostatically triggered and tip-guided, enabled precise displacements over distances exceeding 150 nm, with rotational motion strongly suppressed by bromine substituents that stabilise linear translation.^188^

### Summary

Taken together, the presented studies on large π-conjugated molecules on metallic, graphitic and insulating surfaces highlight the interplay between molecular structure, adsorption geometry, and thermally activated diffusion mechanisms. The diversity of observed behaviours, from simple hopping to long-range, anisotropic motion and tip-induced dynamics, demonstrates that even subtle modifications in molecular architecture or substrate symmetry can alter the energy landscape governing surface mobility and challenging simplified diffusion models. Internal degrees of freedom, conformational flexibility, and cooperative effects such as dimerisation or coordination with adatoms further add to the complexity, underscoring the necessity for multimodal experimental and theoretical approaches.

A systematic understanding of these effects is critical, since polyaromatic and π-conjugated molecules serve as key building blocks in the controlled synthesis of 2D materials and related nanostructures: They offer significant advantages for the synthesis of 2D materials, particularly graphene. Firstly, their stable and flat carbon ring structures facilitate the ordered assembly of 2D carbon, yielding high-quality, low-defect graphene compared to traditional precursors.^189–193^ Secondly, aromatic compounds enable lower growth temperatures in chemical vapour deposition (CVD), increasing energy efficiency and compatibility with temperature-sensitive substrates.^191,194–196^ Thirdly, they are versatile and enable graphene growth across different substrates, including metals such as copper and nickel,^191–193,195,197^ as well as insulators like SiO_2_.^196^ In the field of bottom-up on-surface synthesis of carbon nanostructures, such as 2D conjugated polymers^198^ or graphene nanoribbons,^199^ the dynamics of the initial molecular building blocks used to build such nanostructures is key in the synthesis processes.^200^

Additionally, polyaromatics, such as coronene, allow precise control over layer thickness and uniformity, crucial for tailoring material properties.^192,193,196^ Finally, their efficient decomposition and nucleation kinetics support scalable production, making them promising for industrial applications in electronics, sensors, and composites.^193–195^

At the same time, aromatic compounds containing heteroatoms (*e.g.*, nitrogen, boron, sulfur) offer significant advantages for synthesising doped graphene, which exhibits enhanced electronic, chemical, and catalytic properties.^192,201–205^ Firstly, heteroatom-containing aromatics, such as pyridine or thiophene, enable *in situ* doping during CVD, allowing precise incorporation of heteroatoms into the graphene lattice, thereby modifying its bandgap and conductivity without post-processing.^201,204,205^ Secondly, the molecular structure of heteroatom-containing polyaromatics promotes uniform doping and reduces defect formation, enhancing the quality of doped graphene for applications in transistors, sensors, and energy storage.^201,202^ Finally, as mentioned above the dynamics of C_60_ has been studied and more generally fullerenes constitute another class of π-conjugated molecules which have been employed in electronics as molecular switches,^206^ drug delivery as nanocarriers,^207^ and nanomachines as nanoscale wheels.^208,209^ Their integration with metal-supported graphene exploits Moiré patterns for epitaxial assembly,^210–212^ enhancing structural stability and functional properties. The resulting hybrid materials have been shown to exhibit improved electronic, optical, and chemical characteristics, with applications spanning energy storage, photovoltaics, and catalysis.^213^ The diffusion of fullerenes on graphene and graphite ranges from sliding at low temperatures to rolling at elevated conditions.^214–217^ Recent encapsulation studies further highlight the rich dynamical landscape of fullerenes in confined systems,^218,219^ offering a stable platform to probe molecular transport and to inform the design of next-generation nanoscale devices.

## Conclusion and outlook

The study of surface diffusion of π-conjugated organic molecules has evolved into a multidisciplinary field bridging surface science, physical chemistry, and nanotechnology. This review has outlined how molecular motion on substrates ranging from metals to graphite and 2D materials is governed by a complex interplay of adsorption energetics, surface corrugation, frictional dissipation, and molecular internal degrees of freedom. Experimental and theoretical studies across different molecular classes reveal the sensitivity of nanoscale motion to substrate symmetry and potential energy surface (PES) characteristics: from activated hopping on corrugated metal surfaces to continuous Brownian motion on weakly interacting, flat substrates such as graphite.

General trends in surface diffusion start to emerge. For instance, in benzene, the diffusion barrier decreases with decreasing surface corrugation, leading to faster diffusion on “smoother” substrates and the eventual crossover to Brownian motion. Friction, in contrast, shows less dependence on binding energy and more on molecular geometry and internal molecular dynamics. This is exemplified in five-membered rings where frictional dissipation is dominated by internal rotational modes rather than adsorption strength or mass. Polycyclic systems introduce additional facets, and while ballistic motion has been observed in highly symmetric systems like pyrene on graphite, larger and more anisotropic molecules such as pentacene exhibit anisotropic and thermally activated diffusion.

For larger π-conjugated systems, increasing molecular complexity introduces new internal degrees of freedom and conformational changes to their dynamics, which significantly influence diffusion. The example of cobalt phthalocyanine (CoPc) on Ag(100) illustrates this well: while low-temperature motion proceeds *via* single jumps, higher temperatures give rise to long jumps and a reduced apparent activation barrier. These observations underscore the need for temperature-dependent studies that span the entire regime of the surface processes, an aspect where experimental studies are clearly missing. Such insight is crucial for an accurate determination of diffusion coefficients and, importantly, the often-overlooked prefactor.

Despite substantial progress, important challenges persist. Molecular friction at surfaces remains poorly understood, especially in weakly bound systems where phononic coupling is minimal and long-range interactions or collisions may dominate dissipation. Additionally, current models often neglect or over-simplify internal vibrational and rotational coupling, especially for flexible or functionalised molecules. Advanced machine learning potentials and anharmonic treatments are likely needed to capture such behaviour in greater detail.^220^ Additionally, current experimental techniques face limitations: STM struggles to capture fast dynamics at elevated temperatures, while QENS relies heavily on hydrogen scattering, limiting its applicability to non-hydrogenated systems. Emerging methods, such as machine learning-assisted analysis of scattering data or new time-resolved microscopy approaches, could overcome these barriers and enable real-time observation of multifaceted molecular motion.

In particular the mobility of structurally complex π-conjugated systems, such as functionalised aromatics, large-ring systems like porphyrins, or expanded azahelicenes, remains underexplored despite their relevance in organic electronics, molecular sensors, and responsive interfaces.^31–33,221^ New methodologies, including multiscale modelling and *in situ* spectroscopies, are needed to characterise the potential energy landscapes of these systems.^222–225^ Likewise, the nanoscale motion of aromatic and polyaromatic molecules during CVD processes for crystal growth and 2D material synthesis, as well as in catalytic processes requires further *in situ* characterisation techniques and computational modelling to pave the way for tailored synthesis of 2D materials and functional nanostructures.^44,45,194–196^

The field is also expanding towards more complex substrates, including 2D materials beyond graphene, such as hexagonal boron nitride (h-BN) or vicinal metals,^55,226^ where local electronic structure and topography can drastically reshape the diffusion landscape. Exploring diffusion on diverse substrates such as metal oxides and topological insulators could reveal novel interaction regimes^69,227^ and dedicated intercalation and decoupling of metal-supported 2D materials^228^ offer the prospect to address specific energy dissipation channels in surface diffusion. Furthermore, dynamic and collective phenomena at finite coverages as well as confinement effects in nanostructures give rise to, *e.g.* cooperative motion or dynamic phase transitions, posing new challenges for both experimental characterisation and theoretical modelling.

Beyond equilibrium, non-thermal and externally driven dynamics open new frontiers. Tip-induced motion, photostimulation, or electric field manipulation offer routes for precise control over molecular trajectories, with relevance for molecular machinery and active surfaces.^229^ Such studies also raise foundational questions about energy flow and dissipation under non-equilibrium conditions.^230^

Finally, diffusion studies contribute to adjacent fields, from astrochemistry to environmental effects and the synthesis of novel materials. Beyond terrestrial applications, insights from surface dynamics resonate with astrochemistry. The weak physisorption and low-friction environments of graphitic substrates mirror conditions on carbonaceous grains in interstellar space, where polycyclic aromatic hydrocarbons (PAHs) like pyrene play a key role in prebiotic molecule formation.^52,53,231,232^ In the context of CVD growth molecular diffusion plays a decisive role in nucleation, growth, and interfacial reactivity under elevated temperatures and reactive atmospheres. It influences not only film morphology and grain boundary formation but also governs dopant incorporation and the emergence of functional nanostructures under operando conditions. However, direct studies of diffusion under such dynamic and chemically demanding environments remain scarce. Future investigations are essential to elucidate these processes, ultimately enabling the tailored synthesis of two-dimensional materials with controlled structural and electronic functionalities.^44,45,233,234^

In conclusion, the surface diffusion of π-conjugated molecules constitutes a complex and multifaceted phenomenon shaped by the interplay of molecular structure, substrate characteristics, and external conditions. Advancement in the field will depend on the integration of high-resolution experimental methodologies with sophisticated theoretical and computational approaches, enabling the development of predictive models with relevance to nanofabrication, sensing, catalysis, and related technological applications.

## Conflicts of interest

The authors declare no competing financial interest.

## Data Availability

No primary research results, software or code have been included and no new data were generated or analysed as part of this review.
